# From Congestion to Clarity: On the Complementarity of Resolving Power and Spectral Simplification for Intact Protein Characterization

**DOI:** 10.1021/jasms.6c00016

**Published:** 2026-04-27

**Authors:** Linda B. Lieu, Jingjing Huang, Jake T. Kline, David Bergen, Graeme C. McAlister, Kenneth R. Durbin, Christopher Mullen, Rafael D. Melani, Luca Fornelli

**Affiliations:** † Department of Chemistry and Biochemistry, 6187 University of Oklahoma, Norman, Oklahoma 73019, United States; ‡ 486281 Thermo Fisher Scientific, San Jose, California 95134, United States; § School of Biological Sciences, 6187 University of Oklahoma, Norman, Oklahoma 73019, United States

## Abstract

Top-down mass spectrometry (TDMS) is a powerful platform for the structural and functional analysis of intact proteins, enabling the detailed characterization of proteoforms and precise localization of post-translational modifications. The incorporation of alternative fragmentation techniques, such as electron transfer dissociation, electron transfer higher-energy collisional dissociation, and ultraviolet photodissociation, in instruments such as Tribrid Orbitrap mass spectrometers enhances sequence coverage and improves the confidence in PTM assignment. However, tandem mass spectrometry of intact proteins >30 kDa presents substantial challenges. The resulting spectra are often highly complex with overlapping product ion signals that complicate spectral interpretation. Although increasing the mass resolution can help resolve closely spaced product ions, it is often insufficient to fully alleviate spectral congestion for large proteins. In such cases, proton transfer charge reduction (PTCR) can simplify mass spectra by dispersing product ions across a wider mass-over-charge (*m*/*z*) range. In this study, we evaluated the impact on TDMS of increasing resolving power and PTCR-enabled spectral simplification using four intact proteins: enolase (46.6 kDa), carbonic anhydrase (29 kDa), myoglobin (16.9 kDa), and ubiquitin (8.6 kDa). For carbonic anhydrase, combined MS^2^ fragmentation at low resolving power (60,000 at *m*/*z* 200) yielded 50.5% sequence coverage, which increased to 92.6% at high resolving power (480,000 at *m*/*z* 200) and further to 97.7% when PTCR was applied. This approach was applied to the characterization of biopharmaceuticals by analyzing the three digested and disulfide-reduced ∼25 kDa subunits of the NIST monoclonal antibody (mAb) on a liquid chromatography time scale.

## Graphical abstract

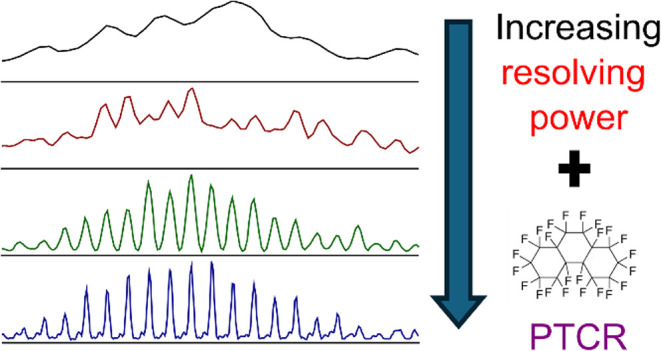

## Introduction

Top-down mass spectrometry (TDMS)^1–3^ is a powerful method for characterizing intact proteins, offering a comprehensive view of post-translational modifications (PTMs), genetic variants, and the structural complexity of entire proteoforms.^4^ A primary goal of TDMS is to generate enough informative product ions during gas-phase dissociation to maximize sequence coverage (defined as the percentage of experimentally observed backbone cleavages relative to those theoretically possible), thereby accurately representing the protein’s primary structure and facilitating the detection and, ideally, the localization of PTMs. Achieving this level of fragmentation requires a dissociation method that induces backbone cleavages in a largely unbiased manner, generating fragments of various sizes with a comparable efficiency.

Various ion dissociation techniques have been employed to increase the extent of backbone bond cleavage in intact protein analysis. Although vibrational excitation techniques usually achieve high conversion of precursor ions into product ions (often defined as the fragmentation efficiency),^5–8^ their preference for cleaving labile bonds and reliance on low-energy dissociation pathways typically limit the generation of diverse and informative fragments, often failing to provide comprehensive sequence coverage for large proteins.^5,9^ Conversely, electron-based dissociation methods such as electron capture dissociation (ECD)^10^ or electron transfer dissociation (ETD)^11^ operate via nonergodic mechanisms, enabling extensive and more uniform backbone cleavages while preserving labile PTMs. These characteristics, combined with their enhanced dissociation efficiency at higher charge states, make electron-based techniques particularly well suited for intact protein analysis.^12–17^ To further increase sequence coverage, hybrid fragmentation strategies have been developed to overcome the formation of nondissociative electron capture/transfer products (ECnoD/ETnoD),^18,19^ which result from persistent noncovalent interactions that prevent product ion separation. For example, ETnoD species can be reactivated within the ion trap using low-energy resonant excitation, a method known as ETcaD.^20^ Alternatively, all ETD products can be transferred to the collision cell for secondary activation via beam-type higher-energy collisional dissociation (HCD), in an approach termed EThcD.^21,22^ In addition to these radical-driven methods, ultraviolet photodissociation (UVPD) using 193 or 213 nm photons has also demonstrated the ability to achieve high sequence coverage in intact protein analysis by accessing multiple backbone cleavage pathways simultaneously.^23–27^

Despite these advancements, attaining complete (e.g., 100%) or near-complete sequence coverage remains increasingly challenging as the protein size grows. The electrospray ionization (ESI) of high-mass analytes produces a distribution of multiple charge states, each of which includes numerous isotopologues.^28^ As the molecular weight increases, both charge state and isotopologue distributions broaden, leading to significant signal dispersion across the mass-to-charge (*m/z)* space. This results in reduced peak intensities and a concomitant decrease in signal-to-noise ratio (S/N), rendering the analysis of proteins larger than 30 kDa significantly more challenging.^29^ Additionally, individual backbone cleavages of multiply charged protein cations are typically represented by product ions with different protonation states, resulting in low-abundance ions characterized by reduced S/N and weak isotopic pattern fidelity.^30^ Finally, when isotopic clusters remain unresolved, confident deconvolution and identification of fragments become highly problematic.^31^ This challenge is further exacerbated when multiple product ions overlap within narrow *m*/*z* regions of a fragmentation spectrum, substantially hindering spectral interpretation and correct product ion assignment.^28^

In Orbitrap Fourier transform mass spectrometry (FTMS), one way to address these issues is by summing time-domain transients prior to Fourier transformation (or alternatively, averaging mass spectra), which improves sensitivity and dynamic range.^32^ This approach mitigates stochastic noise contributions inherent in individual acquisitions, thereby improving the S/N and enhancing confidence in product ion assignment.^33^ Because random noise is uncorrelated across transients, averaging *N* transients reduces noise by a factor proportional to √*N*, resulting in a net S/N improvement of √*N*.^32,34–36^ In FTMS, resolving power (r.p.) increases with transient duration through a linear dependence, since longer acquisition periods allow for finer frequency discrimination.^37^ Finally, S/N improves with the square root of the transient duration,^38–40^ provided there is coherent signal accumulation and minimal ion decay.^35^ The complex signals generated by large, multiply charged proteins provide for time-domain signals that have predictable beat patterns due to the nature of interference (constructive/destructive) between signals arising from various isotope and charge state populations. However, without the acquisition of an additional isotope beat, longer transient acquisitions also begin to introduce additional noise.^41^ And because ion signals decay exponentially during detection, excessively long transients can diminish signal amplitude, effectively reducing S/N.^42^ This introduces a fundamental trade-off between increasing the resolving power and maintaining sufficient S/N, particularly when trying to detect low-abundance species.

To overcome limitations caused by signal overlap and retain sensitivity for low-abundance fragments while still benefiting from high resolving power, gas-phase ion–ion reactions, such as proton transfer reactions^43,44^ can be employed as a complementary strategy. Commercially available as proton transfer charge reduction (PTCR), this reaction shifts product ions to higher *m*/*z* values by reducing their charge states, effectively decongesting spectra by improving the separation of originally overlapping isotopic clusters.^45–48^

In this study, we evaluated the combined impact of data acquisition at high-resolving power and PTCR on the latest-generation Orbitrap Ascend Editions Tribrid mass spectrometer. Using a panel of four standard proteins spanning from 8.6 to 46.6 kDa in mass that were fragmented using a series of orthogonal ion dissociation techniques, we highlight the complementary roles of resolving power and spectral decongestion in improving sequence coverage, particularly for higher-mass proteins. To further assess the practical applicability of combining high-resolving power and PTCR, we extended our analysis to the liquid chromatographic (LC) time scale using ∼25 kDa disulfide-reduced subunits (Fc/2, Lc, and Fd′) of the NIST antibody standard. These experiments underscore the importance of pairing PTCR with high-resolution tandem mass spectrometry (MS^*n*^) for effective characterization of complex proteoforms.

## Materials and Methods

### Sample Preparation

Ubiquitin (8.6 kDa), myoglobin (17 kDa), carbonic anhydrase (29 kDa), and enolase (46.6 kDa) were acquired from Sigma-Aldrich. The proteins were dissolved in a denaturing solution composed of 50% acetonitrile, 25% methanol, 24% water, and 1% formic acid at a 5 μM concentration. NIST monoclonal antibody (mAb) subunits were prepared at a 1 μg/μL final concentration by performing proteolysis with IdeS (Genovis) followed by the reduction of disulfide bonds with dithiothreitol (DTT), according to a previously published protocol.^49,50^

### Mass Spectrometry Data Collection

Data were collected in full-profile mode (i.e., without noise thresholding) on an Orbitrap Ascend BioPharma Tribrid mass spectrometer equipped with IC, ETD, PTCR, UVPD, and Native MS options. Ubiquitin, myoglobin, carbonic anhydrase, and enolase were directly injected using a Thermo Scientific OptaMax NG Ion Source and a syringe pump at 5 μL/min. HCD, ETD, EThcD, and UVPD fragmentation (MS^2^) spectra with and without PTCR (MS^3^) were acquired using different resolving powers of 60,000, 120,000, 240,000, and 480,000. More information regarding the isolated charge states for each protein can be found in Table S1. All nominal r.p. values are calculated at *m*/*z* 200, and we will omit this detail in the rest of the manuscript to improve readability. Subsequent PTCR MS^3^ experiments were collected by reacting whole product ion populations from MS^2^ experiments (using a wide isolation window of 1,800 *m*/*z* units) to PTCR reagents in the linear ion trap for reaction times of 25–50 ms, as previously described.^45,51–53^ Three 1 min technical replicates were acquired for each fragmentation and resolving power combination, with no transient averaging (i.e., one microscan per spectrum). Digested and disulfide-reduced NIST mAb subunits were separated using a Thermo Scientific Vanquish Horizon UHPLC system equipped with a reversed-phase MAbPac column (4 μm particle size, 2.1 mm internal diameter × 50 mm length). Separation was performed over a 16 min gradient, and 1 μg of protein was injected per run. For each run, multiple charge states were quadrupole isolated for fragmenting Lc, Fc/2, and Fd′ using an isolation window of 100 *m*/*z* units centered around *m*/*z* 900 for MS^2^ (Table S1). PTCR MS^3^ experiments were collected in a similar manner to those collected for ubiquitin, myoglobin, carbonic anhydrase, and enolase.

### Data Analysis

Because the number of mass spectra that can be averaged within a minute varies greatly based on resolving power (e.g., r.p. 60,000, ∼450; r.p. 120,000, ∼230; r.p. 240,000, ∼115; r.p. 480,000, ∼55), the number of averaged microscans was standardized across all resolving powers and acquisition parameters (fragmentation method or MS^2^/PTCR MS^3^). For each data file, a single spectrum RAW file was generated by averaging 50 microscans postacquisition in FreeStyle (Thermo Scientific). Manual validation on a single replicate of carbonic anhydrase for each fragmentation technique was conducted using TDValidator (Proteinaceous, Inc.).^54^ The following global settings were used: fragment peak picking with an S/N threshold of 10; fragment mass tolerance set to 10 ppm; interisotopic mass tolerance at 3 ppm; maximum allowed charge state of +25; isotopic peak fitting threshold set to 0.50. Factors such as the qualitative visual consistency in *m*/*z* positions of observed vs theoretical isotopologues, as well as the presence of abundant isotopologue peaks (i.e., at least 3 of the 4 most abundant), were considered during manual validation. The results from the manual validation (Table S2) were used to derive and optimize the autoprocessing parameters in ProSight Native (Proteinaceous, Inc.).^55^ Individual RAW files (available on MassIVE under repository number MSV000099681) were analyzed in batch mode, which enabled the simultaneous processing of multiple files through the integrated TDValidator module. Global settings for batched processing were kept the same as those used for manual validation, with the exception of isotopic peak fitting thresholds, which were set between 0.65 and 0.70 for MS^2^ and 0.55–0.60 for PTCR MS^3^ spectra. Under extensive charge reduction, signal dilution occurs as a single precursor ion is distributed across multiple lower charge states, leading to reduced isotopic fidelity due to limited ion statistics. However, because signal overlap is largely eliminated in PTCR MS^3^ spectra, the confidence in product ion assignment remains high, enabling the use of lower isotopic peak fitting thresholds than in MS^2^. More details on deriving batched processing results can be found in the Supporting Information. For NIST mAb subunits, batched processing was performed as described above, with individual .RAW files generated by averaging mass spectra across the full chromatographic peak of each subunit.

Decoy analyses (i.e., matching of experimental ion clusters against a shuffled protein sequence) were performed using the TDValidator module in ProSight Native on a single replicate of each standard protein. Global search parameters were applied as follows: S/N threshold of 10, fragment mass tolerance of 10 ppm, interisotopic mass tolerance of 3 ppm, maximum charge state of +25, and minimum isotopic peak fitting threshold of 0.60. 1,000 decoy runs were conducted for each experiment. A more detailed description of the decoy analysis is included in the Supporting Information. Plots were generated using GraphPad Prism 9 (GraphPad Software). S/N plots were generated and processed by using an in-house Python script.

## Results and Discussion

### Resolving Power Improves Sequencing in a Protein Mass-Dependent Fashion

The impact of resolving power (r.p.) on protein sequencing was benchmarked using standard proteins spanning in mass from 8.5 to 46.6 kDa.^39^ Following batched processing of all standard proteins, we evaluated the sequence coverages, distributions of mass and charge states, and the number of unique matched product ions across four resolving power values (60,000, 120,000, 240,000, and 480,000) and four fragmentation techniques (ETD, EThcD, HCD, and UVPD). The lowest resolving power, 60,000 (transient duration: 128 ms),^40^ was selected because it reflects the standard setting used for recording fragmentation mass spectra in untargeted top-down proteomics of proteoforms smaller than 30 kDa.^56–58^ Overall, we observed that sequence coverage (Figure S1), and correspondingly, the number of unique matched fragments (Figure S2), increases with higher r.p. across all fragmentation methods. For enolase (Figure S1A), sequence coverage at r.p. 60,000 remained in the low teens or below, regardless of the fragmentation technique used. Among all methods, EThcD consistently produced the highest sequence coverage, reaching 14.5% at r.p. 60,000 and 53.7% at 480,000—a nearly 4-fold increase. Comparatively, coverages were 52.7%, 43.7%, and 42.5% for ETD, HCD, and UVPD MS^2^ experiments at r.p. 480,000, respectively. For carbonic anhydrase, EThcD coverage rose from 37.8% at r.p. 60,000 to 76.0% at r.p. 480,000, representing an approximate 2-fold improvement (Figure S1B).

These results highlight that, particularly for larger proteins (e.g., enolase and carbonic anhydrase), the sequencing potential is not fully realized at lower resolving power, as the lowest coverage was consistently observed at r.p. 60,000. Evidently, the magnitude of improvement strongly correlated with the protein size. The average fold increase in coverage moving from r.p. 60,000 to 480,000 was 4.38, 2.21, 2.15, and 1.20 for protein sizes of 46.6, 29, 16.9, and 8.6 kDa, respectively (Table S3). Notably, the increase in r.p. from 240,000 to 480,000, while smaller, was still apparent for the two largest proteins, reinforcing the trend that larger proteins benefit more from higher resolving power. Further insight came from evaluating the unique fragments obtained at each resolving power using EThcD (selected for this analysis in its capacity as the most effective sequencing method). The number of fragments observed exclusively at r.p. 480,000 decreased progressively across the protein panel: 169 (corresponding to 27.9% of the total across the four r.p. values), 92 (22.8%), 53 (16.4%), and 22 (9.0%) for enolase, carbonic anhydrase, myoglobin, and ubiquitin, respectively (Figures 1 and S3). Inversely, the fraction of fragments shared among all resolving powers increased as the protein size decreased, from 15.8% for enolase to 53.9% for ubiquitin.

**Figure 1:**
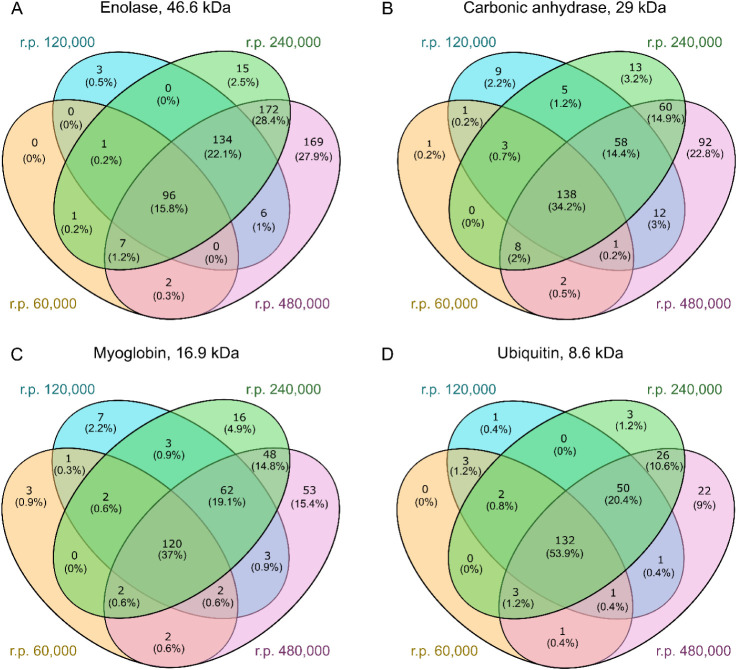
Four-way Venn diagrams of unique fragments found in (A) enolase, (B) carbonic anhydrase, (C) myoglobin, and (D) ubiquitin using EThcD MS^2^ at the four resolving powers. The percentage of shared fragments among all resolving powers is shown to increase as the protein size decreases.

Building on these observations, we next examined fragment mass distributions to determine whether they exhibited similar protein mass-dependent trends. Subtle differences were noted among the fragmentation methods, likely reflecting mechanistic biases in fragment generation (e.g., favoring certain cleavage pathways). However, across all methods, higher resolving powers (≥240,000) consistently improved the distribution toward high-mass fragments (Figures S4). This effect was again most pronounced for the larger proteins (enolase and carbonic anhydrase), whereas for smaller proteins, the distributions began to converge. In the case of ubiquitin (Figure S4, right), fragment mass distributions were nearly identical across fragmentation methods and resolving powers, consistent with the comparable sequence coverage observed under these same conditions (Figure S1). Similarly, when charge state distributions are assessed, the protein mass dependency remains true (Figures S5–S8). In the case of ETD fragmentation of enolase (Figure S5A, left), no charge states above 15+ were detected at r.p. 60,000, while the detection of ions with charge states up to 25+ (the user-defined limit applied during batched processing; note that decoy analyses indicated little to no effect in improving false-positive rates when higher charge limits were used) improved gradually from r.p. 120,000 and beyond. Conversely, for each fragmentation technique, the charge state distributions of ubiquitin fragments look nearly identical among tested r.p. values (Figure S8, left). It is noted that the lack of differences here can be equated to the fact that smaller proteins produce fewer fragments (i.e., have less congested mass spectra) with lower charge states. However, this trend appears to hold primarily for ubiquitin. Carbonic anhydrase and myoglobin demonstrate intermediate behaviors: carbonic anhydrase, being closer in size to enolase, shows more pronounced improvements in fragment charge distribution as a function of r.p. (Figure S6, left), while myoglobin behaves more like ubiquitin, exhibiting relatively modest gains that are between those of ubiquitin and of carbonic anhydrase (Figure S7, left).

### High Resolving Power Is Not Sufficient to Thoroughly Annotate Fragmentation Mass Spectra

Top-down fragmentation of large proteins has still historically suffered from poor S/N, particularly when overlapping signals crowd specific regions of the *m*/*z* space.^59,60^ This limitation is amplified when collecting high resolving power data, since achieving such resolving power in FTMS requires longer transient acquisition times, during which signals from large product ions decay more rapidly than smaller ones due to their greater collisional cross sections.^28,42^ As a result, although higher r.p. can aid in isotopic deconvolution, it only partially offsets the associated losses in sensitivity. In cases of severe spectral congestion, increasing r.p. and thus data acquisition time may provide diminishing returns, making additional strategies essential to disentangle overlapping signals and to enable confident spectral interpretation.

Therefore, we evaluated the complementarity of high r.p. and spectral decongestion using PTCR. In the MS^2^ spectrum of carbonic anhydrase acquired at r.p. 480,000 (at *m*/*z* 200) with EThcD, the majority of product ion signal was compressed into a narrow *m*/*z* ∼1,000 window centered around*m*/*z* 750, while the high boundary of the considered *m*/*z* window was set at *m*/*z* 2,000 (Figure 2A). In contrast, in the applied MS^3^ strategy (which relies on the isolation of all MS^2^-generated products in the linear ion trap by using a single waveform), PTCR induces the deprotonation of all product ions, shifting them to higher *m*/*z* values. This redistribution requires collection over a much broader *m*/*z* range (typically up to *m*/*z* 8,000 on Tribrid Orbitrap instruments),^45,51^ thereby alleviating spectral congestion (Figure 2). The insets shown in panels A and B of Figure 2 display the same zoomed-in region within an MS^2^ spectrum and after PTCR MS^3^ was applied. Only 4 fragment isotopic distributions were matched at the MS^2^ level, albeit with low confidence (poor ion similarity scores, intensity, and S/N, displayed in Table S4). On the other hand, 11 distinct distributions were matched in the PTCR MS^3^ spectrum with substantially higher S/N values (up to 471.2 S/N for fragment *c*_42_^+4^) compared to the sub-50 S/N observed in MS^2^ spectra, demonstrating the benefits derived by the reduction of chemical noise and signal overlap induced by PTCR.

**Figure 2:**
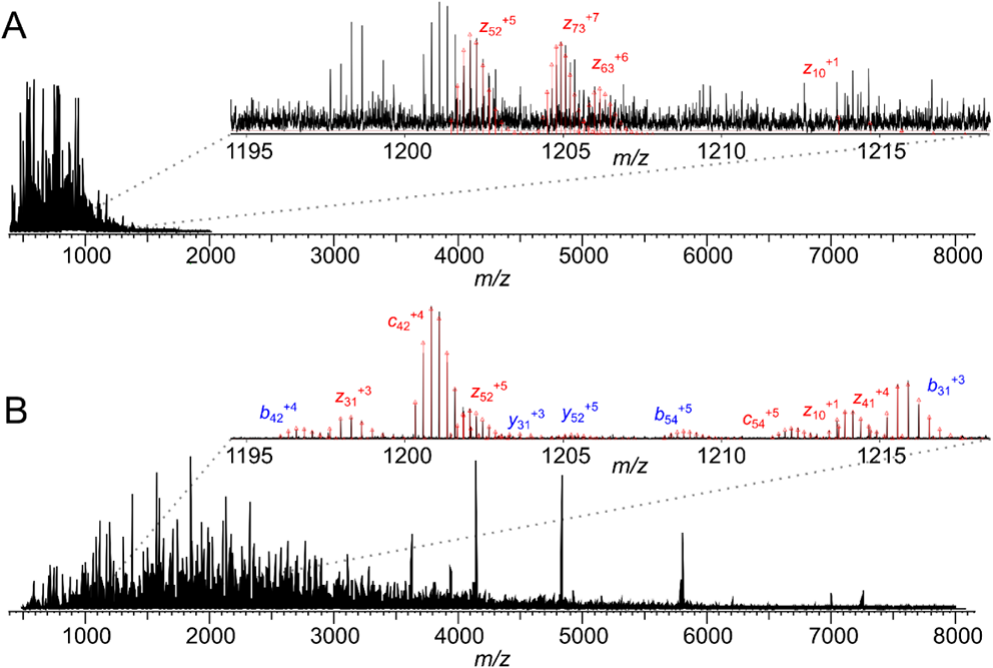
Evaluation of signal dispersion at MS^2^ and PTCR MS^3^ levels. (A) Carbonic anhydrase spectrum collected at r.p. 480,000 with EThcD MS^2^, with the majority of the signal occupying the *m*/*z* 500–1,500 space. (B) Carbonic anhydrase spectrum collected at r.p. 480,000 with EThcD MS^2^ - PTCR MS^3^. The signal is dispersed across a broader *m*/*z* range, so the zoomed-in inset shows distinguishable isotopic distributions.

Decoy analysis of all proteins at both MS^2^ and PTCR MS^3^ levels indicates that PTCR experiments reduce the percentage of expected shuffled fragment matches (i.e., false-positive matches). In TDValidator, decoy analysis is performed by shuffling the proteoform sequence *n* times (user-defined). Each shuffled sequence will have the same length as the original proteoform sequence and is analyzed with the isotopic fitter algorithm using theoretical isotopic distributions generated from the new chemical formula. Although some true-positive matches may still be detected in the decoy analysis due to the presence of isobaric fragments produced by random sequence shuffling, a substantial reduction in false-positive matches is nonetheless observed in the case of PTCR MS^3^ experiments. This trend is consistent across most proteins and fragmentation conditions analyzed, with an average false-positive rate of 11.65% across all MS^2^ experiments, compared to 5.05% for the averaged PTCR MS^3^ experiments (Table S5). This reduction likely reflects the lower fragment density in PTCR spectra; for example, within a *m*/*z* 600–700 window, 61 fragments were matched in MS^2^ spectra compared to only 12 in PTCR MS^3^ spectra (Figure S9).

Furthermore, as expected, the proportion of false-positive matches increases with higher resolving powers—regardless of whether the analysis is performed at the MS^2^ or PTCR MS^3^ level—due to increased signal density and greater spectral complexity as resolving power increases from 60,000 to 480,000. This is also reflected in the number of profile spectrum points (i.e., the individual *m/z–*intensity data points that the TDValidator algorithm uses to search for matched fragments), which increases proportionally with the applied resolving power. For carbonic anhydrase ETD MS^2^ experiments, the number of profile points was 1.84E5, 3.69E5, 7.37E5, and 1.48E6 for r.p. 60,000, 120,000, 240,000, and 480,000, respectively. A similar trend is observed in PTCR experiments, though starting values are higher due to the expanded *m*/*z* range: 2.24E5, 4.47E5, 8.94E5, and 1.80E6. Comparably, larger proteins exhibit higher false-positive matches, as they generally yield more product ions, further exacerbating spectral congestion and the likelihood of random matches during decoy analysis, particularly at higher resolving powers.

Additionally, UVPD on average exhibited higher false-positive rates relative to those of the other fragmentation techniques, especially at the MS^2^ level. Interestingly, for UVPD MS^2^ experiments, the false-positive rate at r.p. 240,000 was occasionally higher than at r.p. 480,000. We hypothesize that this behavior results from the large number of possible product ion types generated by UVPD (nine fragment types were considered in the search), which increases the chance of random matches.

Consequently, PTCR consistently improves sequence coverage and the number of unique matched fragments across all fragmentation techniques and resolving powers when comparing results between MS^2^ and MS^3^ (Figures S10 and S11). The effects of PTCR alone somewhat parallel those of increasing resolving power, where smaller proteins benefit less, as their intact fragmentation generates less signal overlap.^45^ Interestingly, the percentage of fragments shared across all resolving powers increased for all proteins after PTCR, with the exception of carbonic anhydrase (Figure S11). For example, when fragmented via EThcD, ubiquitin exhibited 68.5% shared fragments across PTCR MS^3^ experiments (a 14.6% increase compared to the corresponding MS^2^ results), highlighting that PTCR reduces the dependence on increasing r.p. for unique fragments, particularly for lower-mass proteins. This observation is in agreement with the minimal change in sequence coverage across all tested r.p. values for the ubiquitin data set (Figure S10D). This contrasts with the MS^2^ level, where coverage at r.p. 60,000 was noticeably lower than for the other r.p. values (Figure S1D), and the plateau in sequence coverage only began at r.p. 120,000. Notably, carbonic anhydrase showed a 5% decrease in shared fragments, likely driven by the disproportionately higher number of unique fragments detected at r.p. of 480,000 after PTCR.

Additionally, not only did PTCR MS^3^ experiments exhibit a lower average charge state of fragments compared to their MS^2^ counterparts (Figures S5–S8, right panels), but the charge state distributions also became narrower and less dispersed. This resulted in a transition of the previous broad profiles into more distinct, concentrated peaks, which is a direct consequence of accumulating multiple ions at the same charge state following PTCR. Furthermore, the detection of higher-mass fragments (≥15 kDa) increased in PTCR MS^3^ experiments over MS^2^ ones, along with a rise in overall fragment identifications (Figure 3). In particular, in the enolase ETD MS^2^ experiments performed at r.p. 60,000 and 120,000, only fragments up to 10 and 15 kDa could be detected, respectively. However, PTCR MS^3^ enabled the detection of fragments up to 15 kDa at r.p. 60,000 and up to 20 kDa at r.p. 120,000. Even at higher resolving powers (240,000 and 480,000), where high-mass fragments (≤25 kDa) were already detectable in MS^2^ experiments, implementing PTCR further increased their identification rate (Figure 3B).

**Figure 3:**
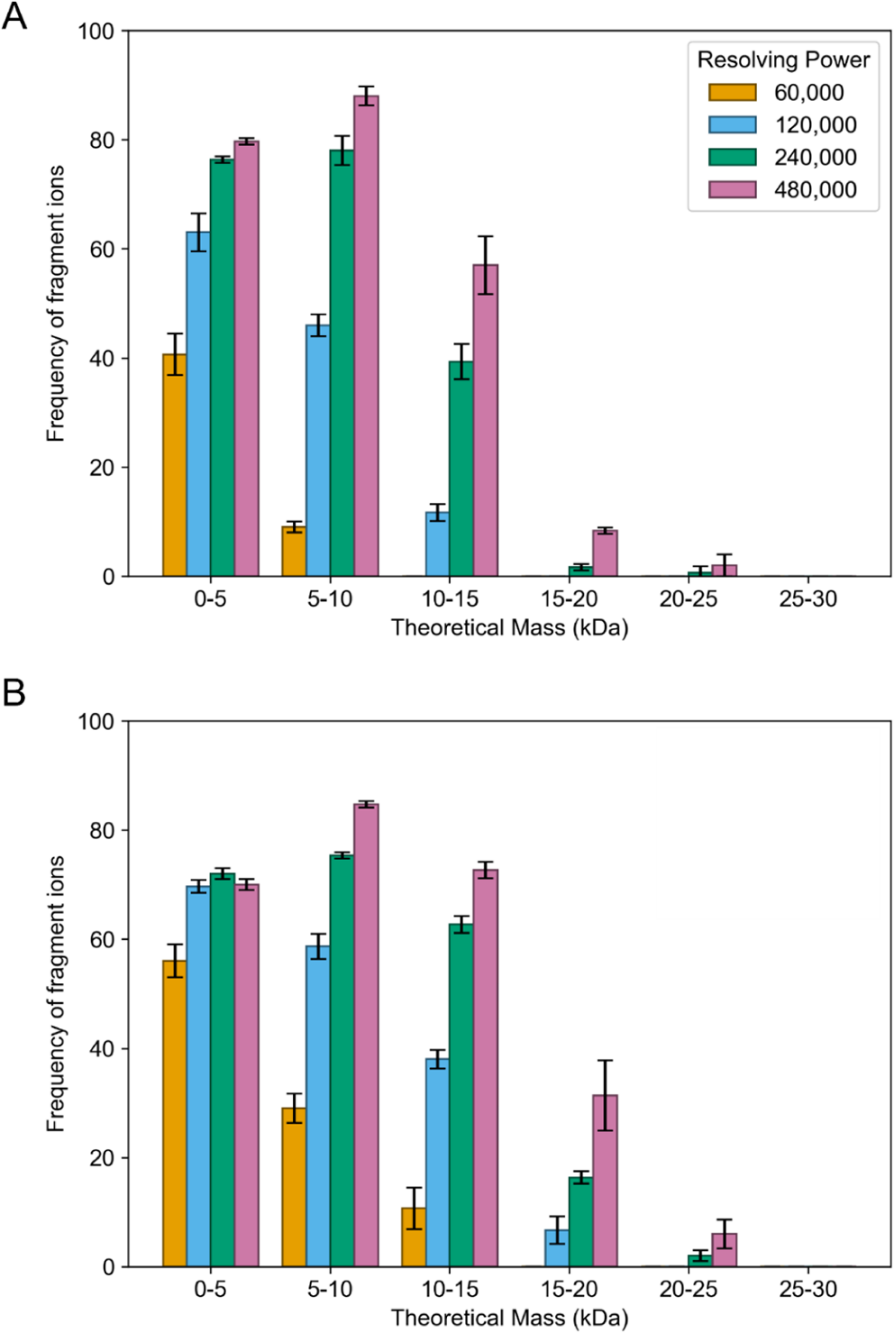
ETD fragment mass distribution of enolase. (A) ETD MS^2^ and (B) ETD MS^2^–PTCR MS^3^ experiments, with three replicates combined to show the mean ± standard deviation. 5 kDa mass bins are used to sort fragment masses detected at the four resolving powers (indicated in different colors as per the figure legend). Performing PTCR MS^3^ facilitates the detection of higher mass fragments (>10 kDa) at all r.p. tested.

The benefits of utilizing r.p. 480,000 are more apparent when examining the enolase data, likely due to the larger mass and subsequent increased complexity of fragmentation spectra. The Venn diagrams in Figure S12 show that r.p. 480,000 yields 147 and 121 unique fragments at the MS^2^ and PTCR MS^3^ levels, respectively. This is substantially more than the 21 and 23 unique fragments observed at r.p. 240,000. Interestingly, more unique fragments are seen at MS^2^ than at the PTCR MS^3^ level. We hypothesize that since data were batch-processed and not manually validated, it is possible that overlapping or ambiguous signals were algorithmically assigned as distinct fragments in MS^2^ spectra. This would be in line with previous studies comparing MS^2^ and corresponding PTCR MS^3^ data sets, which showed that proton transfer reaction leads to a substantial decrease in false-positive fragment assignments.^50,61^ Moreover, inspection of a zoomed-in spectral region reveals that the EThcD mass spectrum collected at r.p. 480,000 enables confident assignment of three additional unique product ions, *c*_99_^+3^, *c*_169_^+5^, and *z*_132_^+4^ over the r.p. 240,000 counterpart (Figure S12B and C).

Further characterizing the contribution of PTCR and resolving power, we next examined their influence on product ion detectability and spectral quality. As shown in Figure 4, there is a clear reduction in spectral noise with increasing resolving power, as visualized in the figure by the red band. Quantitatively, the noise (displayed in the TDValidator software) decreases from 1,398.24 at r.p. 60,000 to 398.75 at r.p. 480,000, resulting in a 3.5-fold reduction and exceeding the theoretical 2.8-fold decrease expected from the r.p. scaling. Moreover, the *c*_186_^+4^ product ion was successfully resolved only by using r.p. 240,000 and 480,000, with S/N of 20.6 and 36.5, respectively. The *b*_186_^+4^ ion was uniquely detected at r.p. 480,000 with an S/N of 18.2. Both fragments are >20 kDa in mass, underscoring the importance of high r.p. for confident assignment of large product ions. Additional advantages of combining high r.p. with PTCR are demonstrated in Figure 5, which compares the combined results of all fragmentation techniques (ETD, EThcD, HCD, and UVPD) at MS^2^ and PTCR MS^3^ levels when applying r.p. 60,000 and 480,000. The combined MS^2^ experiments at r.p. 480,000 yielded 92.6% sequence coverage, compared to 50.5% at r.p. 60,000 (an increase of 1.8-fold). In contrast, the combined PTCR MS^3^ experiments at r.p. 480,000 achieved 97.7% coverage vs 62.8% at r.p. 60,000, representing a 1.5-fold increase. While the relative gain from MS^2^ to PTCR MS^3^ is less pronounced at the highest r.p., the combined effects of PTCR and high r.p. enabled us to approach near-complete sequence coverage. Beyond improvements to overall coverage, we also observed a substantial increase in the number of complementary fragments (i.e., fragments derived from the cleavage of the same backbone bond that retain information regarding the N- and C-terminus) matched in combined PTCR MS^3^ experiments at r.p. 480,000 as compared to MS^2^ experiments collected at the same resolving power, 136 vs 38, respectively (Figure 5C and D). Surprisingly, combined PTCR MS^3^ experiments at r.p. 600,000 did not show the same benefit, yielding 0 complementary fragments compared to the 6 detected at combined MS^2^ experiments at r.p. 60,000. We tentatively attribute this to having used a PTCR duration that shifted large fragments to very high *m*/*z* values, where r.p. 60,000 struggles to baseline resolve isotopologue clusters. This may reflect the inherent reduced complexity of HCD fragmentation spectra when compared against their ETD, EThcD, and UVPD counterparts; therefore, shorter PTCR durations should be applied in order to avoid excessive deprotonation of the original product ions in HCD MS^2^–PTCR MS^3^ experiments. We had slightly reduced the PTCR duration for HCD experiments (i.e., 25 ms for HCD vs 30 ms for ETD); however, further optimization at the lowest r.p. settings may have been needed.

**Figure 4:**
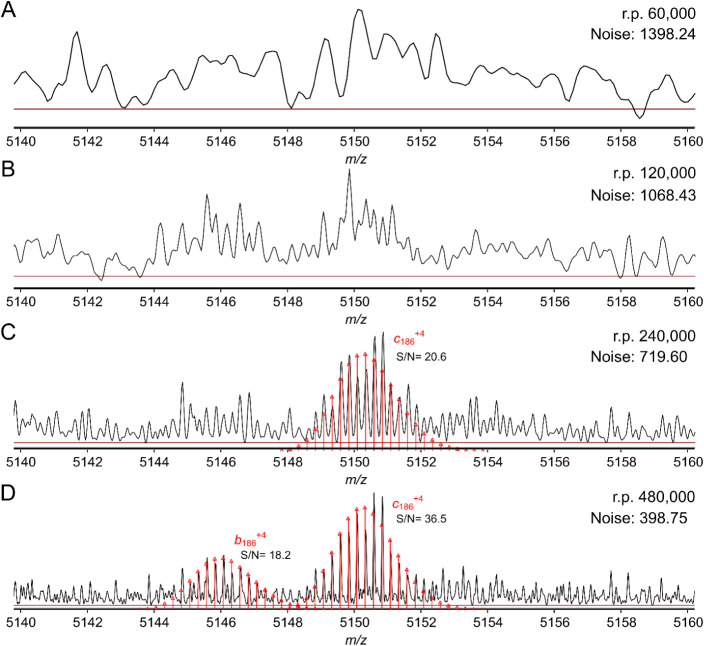
Zoomed-in spectra of EThcD MS^2^–PTCR MS^3^ collected at r.p. (A) 60,000, (B) 120,000, (C) 240,000, and (D) 480,000 for carbonic anhydrase. As r.p. increases, spectral noise visibly decreases. The isotopic pattern of the large product ion *c*_186_^+4^ is clearly distinguished at r.p. 240,000 and 480,000, while an additional *b*_186_^+4^ ion is matched uniquely at r.p. 480,000.

**Figure 5:**
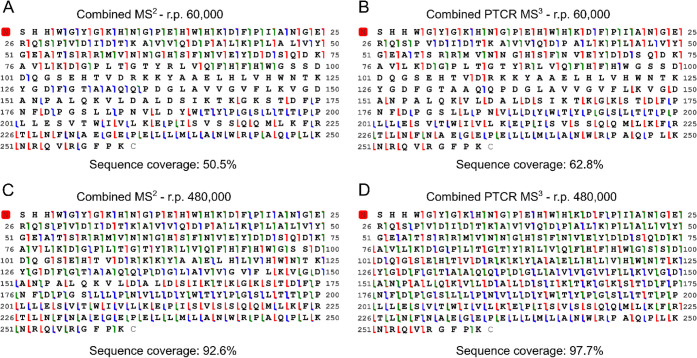
Carbonic anhydrase manually validated fragmentation maps and sequence coverage (%) combining HCD, ETD, EThcD, and UVPD for MS^2^ (left panels), and the combinations with PTCR MS^3^ (right panels) for resolving powers 60,000 (A, B) and 480,000 (C, D). Product ions *a* and *x* are represented by green brackets, *b* and *y* are represented by blue brackets, and *c* and *z* are represented by red brackets.

Next, we examined the experimental dependence of the S/N ratio over resolving power (Figure S13). Consistent with the improved peak definition and reduced baseline interference resulting from the acquisition of longer time-domain transients (Figure 4), a general increase of S/N as a function of r.p. was observed. However, such increases were difficult to assess at the MS^2^ level, especially for enolase and carbonic anhydrase (Figure S14). The S/N values extracted directly from the RAW files using FreeStyle (Thermo Scientific) do not appear to accurately reflect true signal improvements, particularly for large, overlapping product ions. In such cases, the software may be underestimating the noise level or misattributing overlapping ion signals, leading to artificially inflated S/N ratios. Consequently, the S/N values for MS^2^ experiments were capped at 500 to enable visualization in S/N plots. Additionally, any zero and negative values were excluded from the plots (Figure S14). The introduction of PTCR, however, significantly clarifies the spectra, resulting in a measured increase in S/N as a function of r.p. that better correlates with both the theory and the observed improvements in sequence coverage and fragment assignment (Figure S13).^32,62^ The calculated average gain in S/N is not identical across the four tested standard proteins. Moving from r.p. 60,000 to 480,000, ubiquitin displayed an average S/N increase of 2.87, closely aligned with the theoretical value (2.8). Enolase and carbonic anhydrase showed 3.06- and 3.83-fold improvement, respectively. We tentatively attribute these results to potential issues in the correct attribution of noise in fragmentation spectra of larger proteins, as low-abundance isotopologue signals (which could also include neutral losses and internal fragments) may be misinterpreted as chemical noise. Notably, such additional “chemical noise” is more pronounced for very long polypeptide chains. This speculation is partially supported by the observation that in the case of large proteins (i.e., carbonic anhydrase and enolase), the calculated S/N gain surpasses the theoretical value beyond *m*/*z* 2,000, where fragments are more sparse.

To further examine these results, we evaluated the distribution of S/N values across resolving powers without averaging and classified them by the masses of matched fragments. As illustrated in Figure S15, which reports the example of the same fragments *b*_197_ of carbonic anhydrase (21 kDa), detected in HCD MS^2^ and HCD MS^2^–PTCR MS^3^ experiments at increasing r.p., gains in S/N do not linearly scale with increased transient length in the case of MS^2^ data (for which minimal S/N improvement is observed passing from r.p. 240,000 to 480,000), while almost linear improvement is observed in PTCR MS^3^ experiments. A potential explanation of the different behavior observed in the detection of large fragments in MS^2^ and PTCR MS^3^ experiments is that charge reduction may be accompanied by a reduction of collisional cross section (CCS), which in turn would improve the half-life of ions so that their signal can be recorded for a longer period of time. Several studies have determined that low ion charge states, regardless of whether they are obtained by directly ionizing a polypeptide under native conditions or by deprotonating an originally denatured cation using proton transfer reactions, are associated with reduced CCS compared to a more highly charged state.^63–65^ This observation was confirmed with the analysis of all ETD-generated fragments identified through batched processing for carbonic anhydrase (Figure S16). Moving from r.p. 240,000 to 480,000 does not necessarily provide a significant S/N improvement for large fragments in the MS^2^ experiment. In contrast, this effect is much more evident in PTCR MS^3^ data, where spectra acquired at r.p. 480,000 show higher identification numbers across both low- and high-mass bins together with improved S/N.

### Effect of r.p. and PTCR on the Characterization of Antibody Subunits on the Chromatographic Time Scale

To demonstrate the capabilities of high resolving power and PTCR for the analysis of biotherapeutics, we measured IdeS-digested, disulfide-reduced NIST mAb subunits under the same conditions as the standard top-down proteins. Subunits were separated via reversed-phase liquid chromatography (LC), which constrains the number of spectra acquired across each elution peak. This effect is further compounded at higher r.p., where longer transient acquisition times will reduce the number of transients per elution peak. Because all available spectra are typically averaged to maximize sensitivity and faithfully capture the elution profile, the transient counts were not the same across different r.p. in this context. That said, we achieve results similar to those of the standard proteins when implementing high resolving power with PTCR at the chromatography time scale. In particular, applying PTCR increases sequence coverage across nearly all subunits and conditions (Table S6). When the changes in coverage, both the gains and losses, were averaged, the overall improvement following PTCR was 7.6% (Table 1).

**Table 1:** Sequence Coverage Differences on NIST mAb Subunits between PTCR MS^3^ and MS^2^ for Data Acquisition^a^ ^b^

Sequence coverage difference between PTCR MS^3^ and MS^2^ data					
Subunit	Fragmentation technique	60k	120k	240k	480k
Fc/2	ETD	11.3	8.7	5.2	2.2
	EThcD	11.0	15.8	27.2	13.4
	HCD	1.2	–3.4	–5.3	–8.6
	UVPD	4.8	0.3	10.5	3.8
Lc	ETD	7.6	6.6	11.1	7.8
	EThcD	11.1	13.7	19.6	18.7
	HCD	5.4	0.3	–2.8	3.6
	UVPD	3.1	6.8	8.5	13.0
Fd′	ETD	3.2	11.8	12.0	7.4
	EThcD	8.5	10.7	27.1	16.2
	HCD	2.4	–0.8	13.7	10.3
	UVPD	–0.3	1.7	5.5	2.2

a Positive values indicate an improvement in sequence coverage with PTCR MS^3^, expressed as the percentage point increase.

b Negative values indicate a decrease in the coverage.

We then evaluated whether the additional 512 ms of transient time necessary for doubling r.p. from 240,000 to 480,000 offers a meaningful advantage at the chromatographic time scale. At the MS^2^ level, increasing the resolving power resulted in an average improvement of 6.81 in sequence coverage across all fragmentation techniques and all three subunits (Table S6). Conversely, the sequence coverages showed a more modest increase between the two r.p. settings at the PTCR MS^3^ level (Figure S17), resulting in a net improvement of 3.28 (Table S6). Although EThcD MS^2^–PTCR MS^3^ experiments showed an average decrease in coverage at r.p. 480,000, particularly for Fc/2 and Fd′ subunits, the variability indicated by the error bars suggests that these differences may not be substantially meaningful, and the overall performance for the two r.p. settings is effectively similar (Table S6, Figure S17). The Lc subunit likewise showed only a modest increase when PTCR was applied. Consistent with this interpretation, analysis of the Fc/2 subunit revealed that the majority of matched fragments were shared between both data sets (236, or 79% of the total), while a similarly small number were unique to either data set (32 at r.p. 240,000 vs 29 at r.p. 480,000), as shown in Figure S18. The few fragments detected only at r.p. 240,000 that were not algorithmically identified at r.p. 480,000 likely reflect the reduced number of averaged spectra.

Overall, while r.p. of 480,000 generally affords higher sequence coverage, particularly at the MS^2^ level, the added benefits become more diminished at the PTCR MS^3^ level. For the ∼25 kDa subunits examined here, when effective fragmentation techniques such as EThcD were employed, r.p. 240,000 was sufficient to achieve near-maximal coverage, especially after applying PTCR. However, higher resolving powers may still be warranted for larger proteins or complexes.^57^ Thus, the optimal resolving power ultimately depends not only on chromatographic constraints but also on analyte size and the selected fragmentation method.

## Conclusions

These results demonstrate that acquiring fragmentation data at increasing resolving power enhances overall sequencing metrics at both the MS^2^ and PTCR MS^3^ levels for larger proteins (>25 kDa), while having a more limited impact on smaller proteins (<10 kDa). However, performing PTCR is particularly important when acquiring data at high resolving power for large proteins, as spectral congestion can otherwise diminish the benefits of longer transients.

While combining complementary fragmentation methods (ETD, EThcD, HCD, and UVPD) can further increase sequence coverage (Figure 5), the additional benefit becomes relatively modest when high resolving power and PTCR are already employed. For carbonic anhydrase, sequence coverage increased from approximately 88% for a single EThcD MS^2^–PTCR MS^3^ acquisition at r.p. 480,000 to 97.7% after integrating data from all four fragmentation techniques—a gain of only about 10%, despite requiring roughly four times longer acquisition times. Moreover, when considering comparable acquisition efficiency, such as the combined methods of r.p. 60,000 (equivalent to 1/4 of the time of an r.p. 480,000 acquisition) vs a single PTCR acquisition at r.p. 480,000, the latter still provides higher sequence coverage (62.8% combined vs 88%, respectively). This suggests that once resolving power and PTCR conditions are optimized, the marginal benefits of combining multiple fragmentation modes diminish relative to the associated increase in acquisition time.

Combining high resolving power with PTCR can enable near-complete sequence coverage, at least for proteins <30 kDa, and as the top-down proteomics community continues to pursue the characterization of larger and more complex proteins, the strategies outlined here can serve as a framework for optimizing acquisition parameters.

## Supplementary Material



## Notes

Supporting figures and tables include the charge state and *m*/*z* selected for each protein fragmentation technique; comparison between manually validated and batch-processed results; MS^2^ batched processing results indicating fold-change when increasing resolving power; decoy analysis for MS^2^ and PTCR MS^3^ experiments for standard proteins; signal-to-noise ratio and similarity score value of fragments observed in zoomed-in sets of Figure 2; full summary sequence coverage of all NIST experiments; MS^2^ sequence coverage summary of enolase, carbonic anhydrase, myoglobin, and ubiquitin; MS^2^ unique fragment ion count summary of enolase, carbonic anhydrase, myoglobin, and ubiquitin; percentage of unique and shared fragment ions observed at different resolving powers; fragment mass violin plots for the standard proteins; charge state distribution violin plots for enolase at MS^2^ and PTCR MS^3^ experiments; charge state distribution violin plots for carbonic anhydrase at MS^2^ and PTCR MS^3^ experiments; charge state distribution violin plots for myoglobin at MS^2^ and PTCR MS^3^ experiments; charge state distribution violin plots for ubiquitin at MS^2^ and PTCR MS^3^ experiments; comparison of fragment ion density between MS^2^ and PTCR MS^3^ experiments of manually validated carbonic anhydrase ETD data; PTCR MS^3^ sequence coverage summary of enolase, carbonic anhydrase, myoglobin, and ubiquitin; 4-way Venn diagram of unique fragments found in the standard proteins using EThcD MS^2^–PTCR MS^3^ at the four resolving powers; comparison of unique EThcD fragments identified at different resolving powers in Enolase protein; average signal-to-noise ratio distribution across the *m*/*z* space for the standard protein in ETD MS^2^–PTCR MS^3^ experiments; average signal-to-noise ratio distribution across the *m*/*z* space for the standard protein in ETD MS^2^ experiments; isotopic distribution and signal-to-noise ratio of *b*_197_ fragment from carbonic anhydrase at HCD MS^2^ and PTCR MS^3^ experiments at various resolving powers; scatterplot of product ion signal-to-noise values grouped into 5 kDa wide mass bins for MS^2^ and PTCR MS^3^; sequence coverage of NIST mAb subunits; comparison of unique EThcD fragments identified at different resolving powers for NIST Fc/2 subunit (PDF)
