# Supplementary material for: From Congestion to Clarity: On the Complementarity of Resolving Power and Spectral Simplification for Intact Protein Characterization
Source: J Am Soc Mass Spectrom. 2026 Apr 27;37(5):1226–36. doi: 10.1021/jasms.6c00016 (PMC13154342; doi:10.1021/jasms.6c00016)
Supplement: Supplementary file 1 [file js6c00016_si_001.pdf]

SUPPORTING INFORMATION

From congestion to clarity: on the complementarity of resolving power and spectral simplification for intact protein characterization

Linda B. Lieu,[1] Jingjing Huang,[2] Jake T. Kline,[3] David Bergen,[2] Graeme C. McAlister,[2] Kenneth R. Durbin,[2] Christopher Mullen,[2] Rafael D. Melani,[2] Luca Fornelli[1,3*]

[1]*Department of Chemistry and Biochemistry, University of Oklahoma, Norman, OK, 73019 USA*

[2]*Thermo Fisher Scientific, San Jose, CA, 95134 USA*

[3]*School of Biological Sciences, University of Oklahoma, Norman, OK, 73019 USA*

* to whom correspondence should be addressed: School of Biological Sciences, Richards Hall 411b, 730 Van Vleet Oval, Norman, OK, 73019. Phone: 405-325-1483; fax: 405-325-6202; email: luca.fornelli@ou.edu

Table of Contents

Table S1 3
Table S2 4
Table S3 5
Table S4 6
Table S5 7
Table S6 8
Figure S1 9
Figure S2 10
Figure S3 11
Figure S4 12
Figure S5 13
Figure S6 14
Figure S7 15
Figure S8 16
Figure S9 17
Figure S10 18
Figure S11 19
Figure S12 20
Figure S13 21
Figure S14 22
Figure S15 23
Figure S16 24
Figure S17 25
Figure S18 26

Table S1. Charge state and *m/z* selected for each protein fragmentation technique

Protein	Frag method	*m/z*	Charge state
Enolase	ETD	741.8	63
	EThcD	741.8	63
	HCD	1112	42
	UVPD	1262	37
Carbonic anhydrase	ETD	745.2	39
	EThcD	745.2	39
	HCD	1210	24
	UVPD	1210	24
Myoglobin	ETD	771.5	22
	EThcD	771.5	22
	HCD	998.1	17
	UVPD	998.1	17
Ubiquitin	ETD	714.6	12
	EThcD	714.6	12
	HCD	857.5	10
	UVPD	1072	8
NIST IgG1: Fc/2	ETD	902	28
	EThcD	902	28
	HCD	902	28
	UVPD	902	28
NIST IgG1: Lc	ETD	926	25
	EThcD	926	25
	HCD	926	25
	UVPD	926	25
NIST IgG1: Fd'	ETD	918	28
	EThcD	918	28
	HCD	918	28
	UVPD	918	28

	r.p. 60,000				r.p. 480,000			
	MS2		PTCR MS3		MS2		PTCR MS3	
Fragmentation technique	Manual validation	Batched	Manual Valdiation	Batched	Manual validation	Batched	Manual Valdiation	Batched
ETD	37.6	34.2	41.9	43.3	73.6	73.5	87.2	87.9
EThcD	38.4	37.8	43.0	43.6	75.6	76.0	88.8	88.5
HCD	20.9	22.3	15.9	16.4	50.4	51.1	54.3	56.6
UVPD	26.4	25.1	18.6	18.2	60.1	60.3	69.8	70.2

Table S2. Comparison between manually validated and batch-processed sequence coverage of carbonic anhydrase at r.p. 60,000 and 480,000 in MS^2 and PTCR MS^3 experiments. All fragmentation methods were manually analyzed using TDValidator with consistent global settings: an isotopic peak fitting score threshold of 0.5, fragment mass tolerance of 10 ppm, and S/N cutoff of 10. The manually validated results informed the parameter selection for subsequent batched analysis in PSNative. The score threshold was varied between 0.65-0.70 for MS^2 and 0.55-0.60 for PTCR MS^3 experiments in batched-processing to closely match the sequence coverage achieved from manual validation. In addition, the inter-isotopologue tolerance was adjusted to 2.0-3.0 ppm to closely match manual validation results. Generally, r.p. 480,000 required stricter parameters due to a substantial increase in the number of detected peaks, especially for larger proteins. The batched processing results represent an average of three replicates, and the settings used closely resemble the results obtained from manual validation.

Protein size	Fragmentation Technique	60k:120k	120k:240k	240k:480k	60k:480k	Average
46.6 kDa	ETD	2.45	1.62	1.17	4.65	2.47
	EThcD	2.28	1.33	1.23	3.71	2.14
	HCD	1.67	1.59	1.39	3.67	2.08
	UVPD	2.47	1.99	1.13	5.52	2.78
29 kDa	ETD	1.60	1.19	1.13	2.15	1.52
	EThcD	1.49	1.23	1.10	2.01	1.46
	HCD	1.44	1.31	1.22	2.29	1.56
	UVPD	1.62	1.32	1.12	2.40	1.62
16.9 kDa	ETD	1.78	1.10	1.05	2.06	1.50
	EThcD	1.43	1.12	1.06	1.70	1.33
	HCD	1.47	1.42	1.15	2.41	1.61
	UVPD	1.75	1.32	1.05	2.44	1.64
8.6 kDa	ETD	1.06	1.00	1.03	1.10	1.05
	EThcD	1.09	1.02	1.01	1.13	1.06
	HCD	1.09	1.05	1.00	1.15	1.07
	UVPD	1.30	1.08	1.00	1.40	1.19

Table S3. Batched processing results showing the fold change in MS^2 sequence coverage for standard proteins, listed in order of decreasing molecular weight: Enolase, carbonic anhydrase, myoglobin, and ubiquitin. In general, sequence coverage improves as resolving power is doubled, but the extent of improvement diminishes with decreasing protein size. For ubiquitin, little improvement was observed, with an average fold change of 1.

Fragment name	S/N	Fragment score
MS^2 fragments		
z_{10}^{+1}	6.7	0.91
z_{52}^{+5}	39.7	0.8
z_{63}^{+6}	18.5	0.71
z_{73}^{+7}	36.9	0.7
MS^3 PTCR fragments		
b_{31}^{+3}	195.7	0.88
b_{42}^{+4}	39.1	0.8
b_{54}^{+5}	29.4	0.79
c_{42}^{+4}	471.2	0.91
c_{54}^{+5}	39.2	0.83
y_{31}^{+3}	18.3	0.84
y_{52}^{+5}	15.4	0.81
z_{10}^{+1}	46	0.84
z_{31}^{+3}	80.4	0.9
z_{41}^{+4}	98.2	0.95
z_{52}^{+5}	103	0.88

Table S4. Signal-to-noise ratio and similarity score values of fragments observed in zoomed-insets of Figure 2, representing MS^2 and PTCR MS^3 fragmentation experiments of carbonic anhydrase based on EThcD.

Protein	Fragmentation technique	60k		120k		240k		480k	
		MS2	MS3	MS2	MS3	MS2	MS3	MS2	MS3
Enolase	ETD	2.6	1.7	4.6	1.8	17.7	2.5	21.8	6.9
	EThcD	4.2	3.7	8.1	2.9	23.7	4	25	10.9
	HCD	3.8	5.2	8.5	4.1	19.5	9.2	22.4	10.9
	UVPD	8.7	3.1	20.7	6.7	42.7	13	27.9	17.6
Carbonic Anhydrase	ETD	1.3	0.7	3.5	1.2	14	2.3	20.9	4.5
	EThcD	2.6	1.3	6.3	2.1	21.4	4.2	25	7.2
	HCD	2.6	0.9	4.1	3.2	5.3	4.6	18.1	6.7
	UVPD	5.1	2.1	16.3	4.3	30.1	10.8	29	15.1
Myoglobin	ETD	2.2	1.2	4.7	1.8	10.2	2.8	16.3	3.9
	EThcD	3.6	2	8	3.3	14.7	4.5	25.2	6.1
	HCD	4.9	1	5.2	4.4	8.4	5.1	14.8	6.6
	UVPD	11.4	4.3	11.4	8.7	13.3	9.3	23.9	10
Ubiquitin	ETD	2.2	2.6	3	3.3	4.4	3.7	6.3	4.5
	EThcD	3.5	4.3	4.7	4.7	6.2	5.2	8.2	6.4
	HCD	2.5	1.7	3.8	2.1	5.1	3	7	3.9
	UVPD	8	6.5	9.2	6.2	11.2	7.2	14.4	7.8

Table S5. Decoy analysis for MS^2 and PTCR MS^3 experiments for standard proteins. A proteoform spectral decoy search was performed as follows. Protein sequences were shuffled 1000 times. Each shuffled sequence, with the same sequence length as the originating protein, was analyzed with the isotopic fitter algorithm using theoretical isotopic distributions generated from its new chemical formula. The number of matching fragment ions against the input spectrum was found for each instance. The mean of these matching fragment values was then calculated and converted into a percentage that provides the proportion of the overall matching fragment ions that might be expected to match with any sequence of the same length as the proteoform given the spectrum.

		MS2				PTCR MS3			
Subunit	Fragmentation technique	60k	120k	240k	480k	60k	120k	240k	480k
Fc/2	ETD	22.25 ± 0.75	44.45 ± 3.35	56.0 ± 3.8	65.3 ± 1.7	33.5 ± 0.5	53.1 ± 2.4	61.2 ± 0	67.45 ± 3.85
	EThcD	24.2 ± 1.2	39.25 ± 1.45	52.2 ± 0	63.15 ± 1.45	35.15 ± 0.75	55 ± 0.5	79.4 ± 0.5	76.55 ± 2.35
	HCD	12.2 ± 0.2	24.4 ± 0.5	30.4 ± 0.7	39.0 ± 3.6	13.4 ± 0.5	21.05 ± 3.35	25.1 ± 0.7	30.4 ± 1.7
	UVPD	16.75 ± 0.45	35.4 ± 1.0	35.4 ± 1.0	51.7 ± 0.5	21.55 ± 0.95	35.65 ± 0.25	45.9 ± 1.9	55.5 ± 2.4
Lc	ETD	24.55 ± 0.45	46.95 ± 2.15	53.75 ± 0.45	57.3 ± 1.2	32.1 ± 0	53.55 ± 1.15	64.85 ± 0.25	65.1 ± 1.9
	EThcD	24.5 ± 1.9	51.15 ± 1.65	58.5 ± 0	63.2 ± 0	35.6 ± 1.2	64.85 ± 3.55	78.05 ± 1.65	81.85 ± 1.65
	HCD	18.65 ± 0.25	32.55 ± 0.95	37.5 ± 0.7	37.5 ± 0.7	24.05 ± 0.45	32.8 ± 1.2	34.7 ± 0.7	41.05 ± 1.85
	UVPD	21.45 ± 1.15	32.1 ± 0	44.8 ± 1.9	51.4 ± 1.4	24.55 ± 0.45	38.9 ± 2.1	53.3 ± 0	64.4 ± 0.7
Fd	ETD	16.4 ± 0.4	36.8 ± 0.2	44.75 ± 1.45	51.45 ± 1.05	19.55 ± 0.65	48.55 ± 0.65	56.75 ± 1.25	58.85 ± 1.25
	EThcD	17.4 ± 0.2	41.2 ± 0	48.3 ± 0	57.75 ± 0.65	25.85 ± 3.15	51.85 ± 1.05	75.4 ± 0.2	73.95 ± 1.65
	HCD	11.95 ± 0.65	26.8 ± 2.9	23.1 ± 1.3	27.95 ± 0.65	14.3 ± 0.4	26.05 ± 2.15	36.75 ± 3.55	38.25 ± 3.35
	UVPD	18.3 ± 1.9	28.15 ± 0.85	36.15 ± 0.85	36.95 ± 0.85	18.05 ± 0.85	29.85 ± 0.85	41.6 ± 2.5	39.1 ± 0.4

Table S6. Full summary sequence coverage of all NIST experiments. Each result represents the average of 2 replicates.

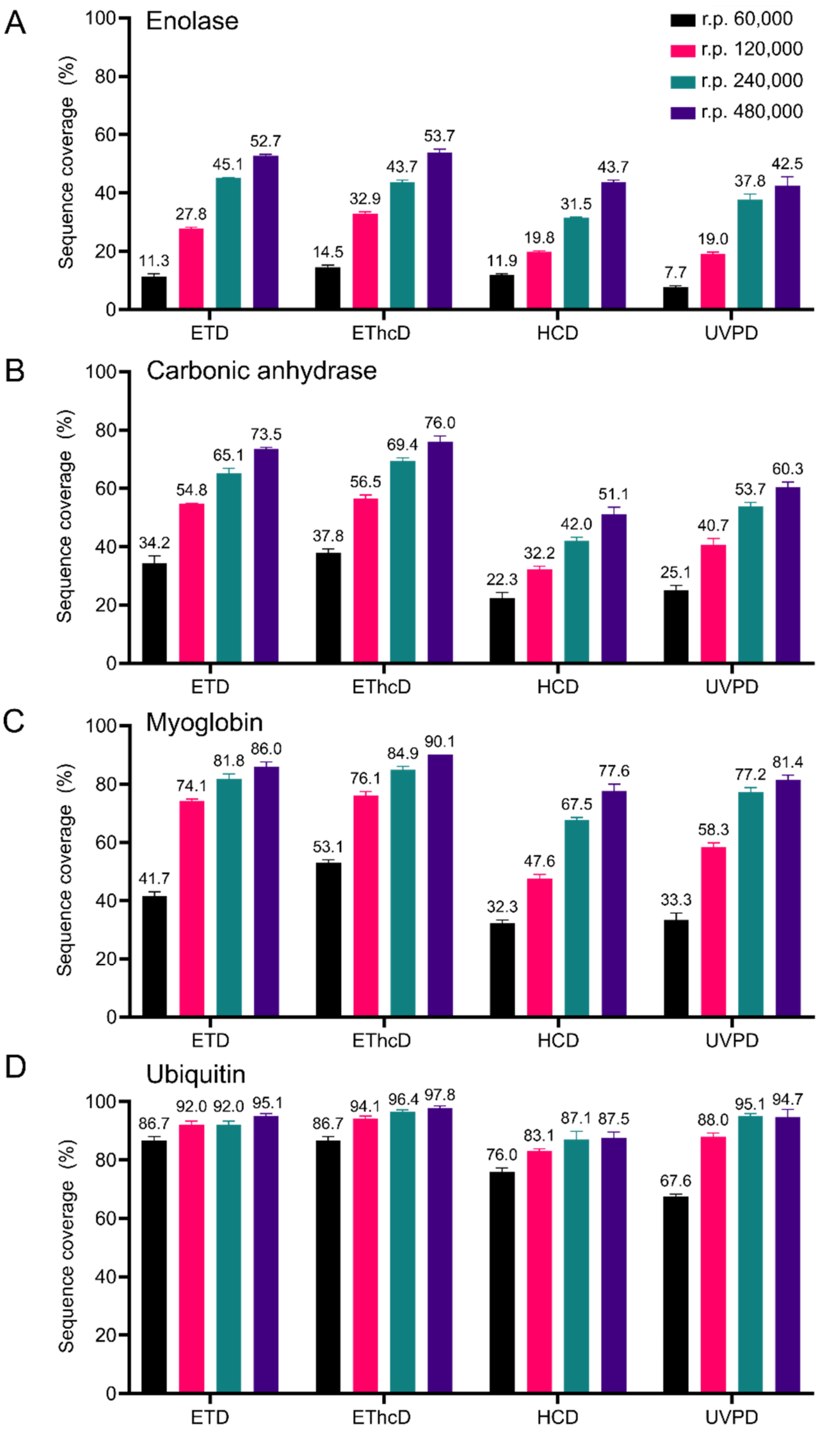

Figure S1. Sequence coverage of (A) Enolase, (B) Carbonic anhydrase, (C) Myoglobin, and (D) Ubiquitin MS^2 experiments at 60,000, 120,000, 240,000, and 480,000 resolving power in the colors black, pink, green, and purple, respectively. Proteins are arranged in order of molecular weight, with the largest on top (Enolase, 46.6 kDa) and the smallest on the bottom (Ubiquitin, 8.6 kDa).

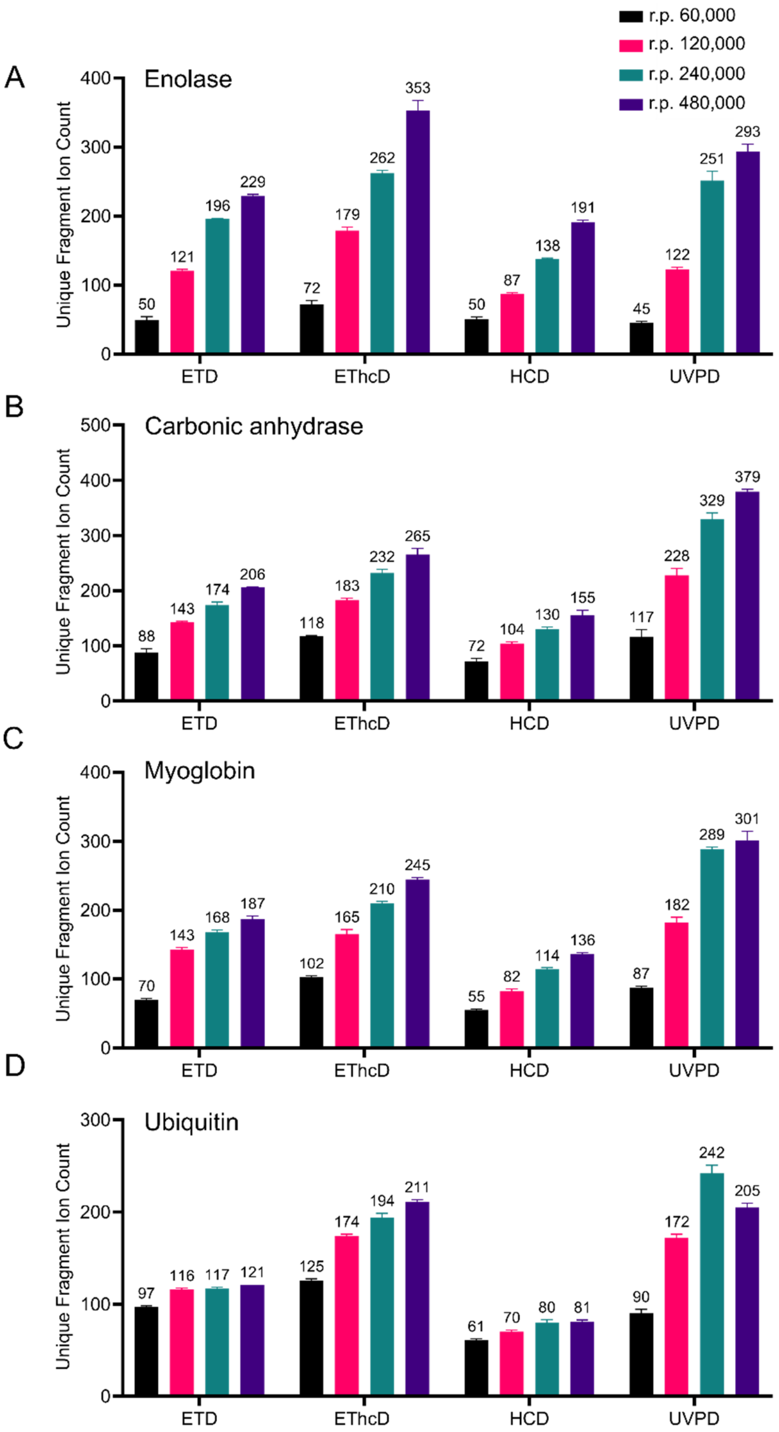

Figure S2. Unique fragment ion count of (A) Enolase, (B) Carbonic anhydrase, (C) Myoglobin, and (D) Ubiquitin MS^2 experiments at 60,000, 120,000, 240,000, and 480,000 resolving power in the colors black, pink, green, and purple, respectively. Proteins are arranged in order of molecular weight, with the largest on top (Enolase, 46.6 kDa) and the smallest on the bottom (Ubiquitin, 8.6 kDa).

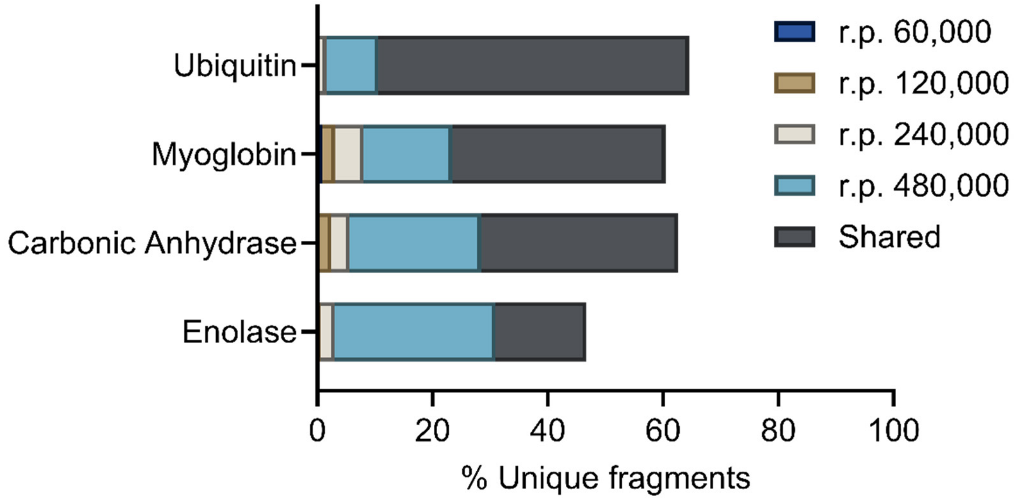

Figure S3. Percentage of unique and shared fragment ions observed at different resolving powers. The plot shows the percentage of fragments uniquely detected at each resolving power alongside the overall percentage of fragments shared across all resolving powers. As protein size increases, the proportion of unique fragments detected at a resolving power of 480,000 increases, while the percentage of fragments shared across all resolving powers decreases. This representation provides a simplified overview of the resolving power trends originally illustrated in Figure 1.

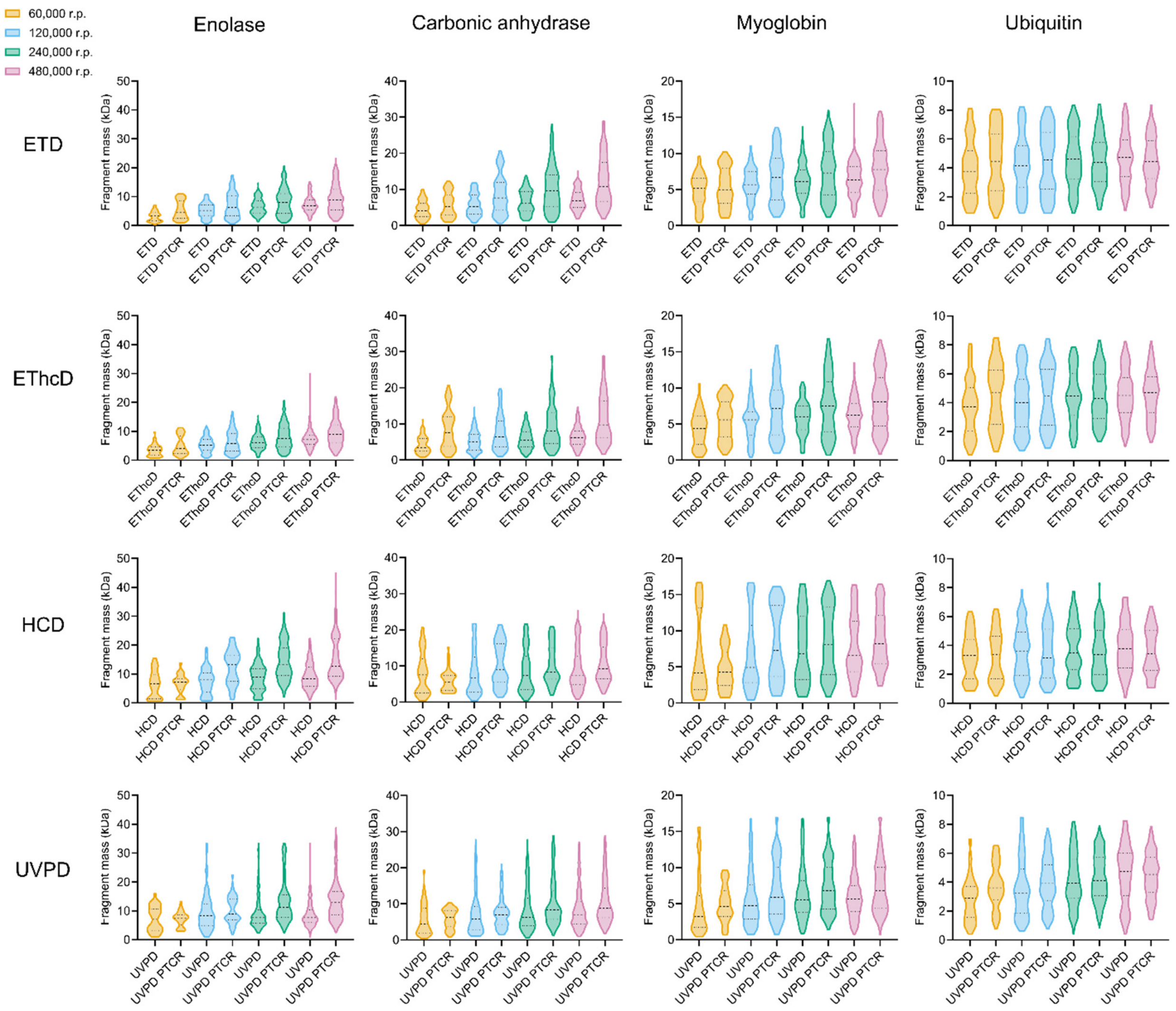

Figure S4. Enolase, carbonic anhydrase, myoglobin, and ubiquitin fragment mass violin plots under ETD, EThcD, HCD, and UVPD fragmentation conditions at 60,000 , 120,000 , 240,000 , and 480,000 resolving powers for MS^2 and PTCR MS^3.

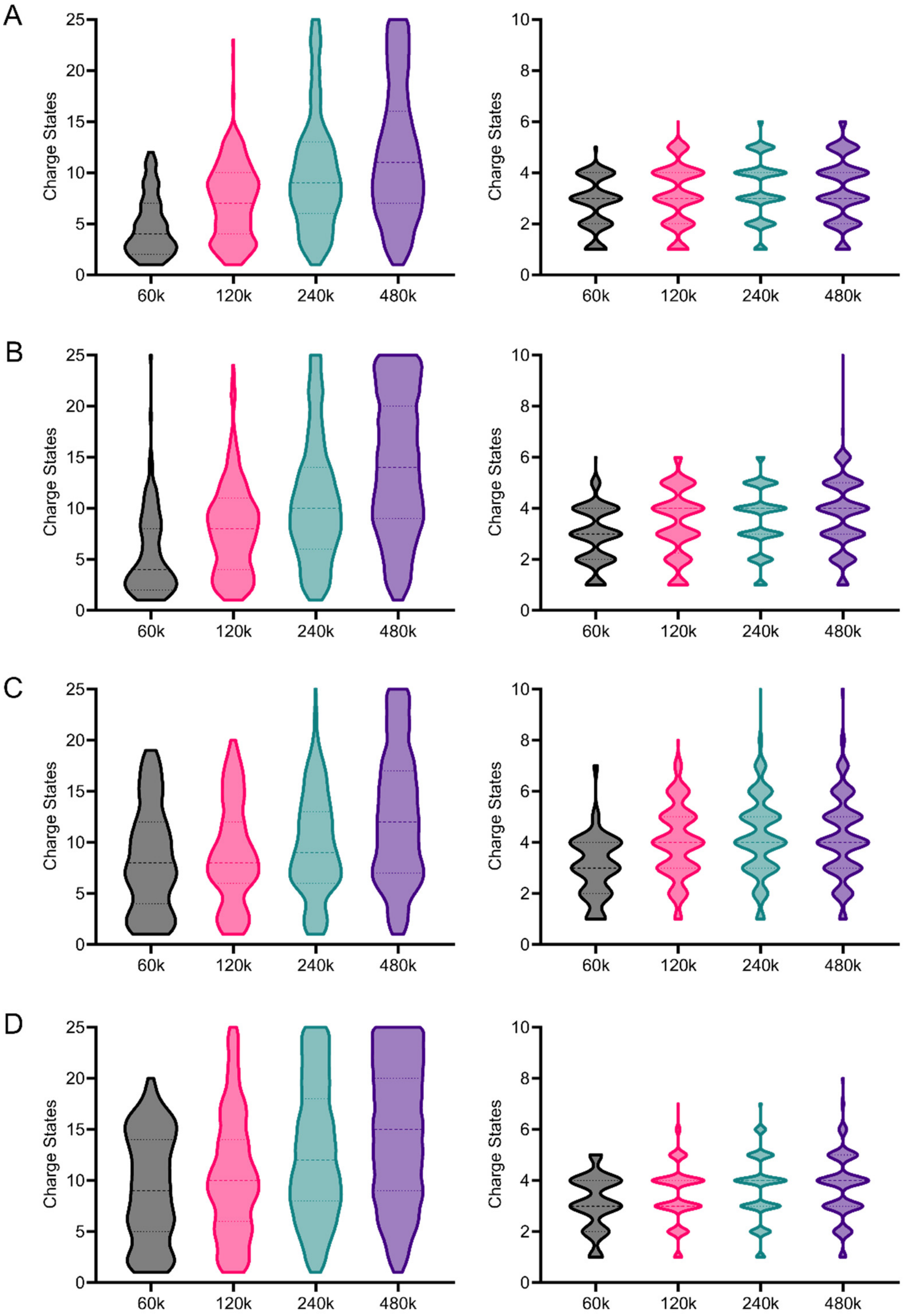

Figure S5. Charge state distribution violin plots of Enolase for (A) ETD, (B) EThcD, (C) HCD, and (D) UVPD fragmentation techniques. Each panel compares MS^2 (left) and PTCR MS^3 (right) results. Experiments performed at higher resolutions (240,000 in green and 480,000 in purple) exhibit a greater number of high charge state identifications.

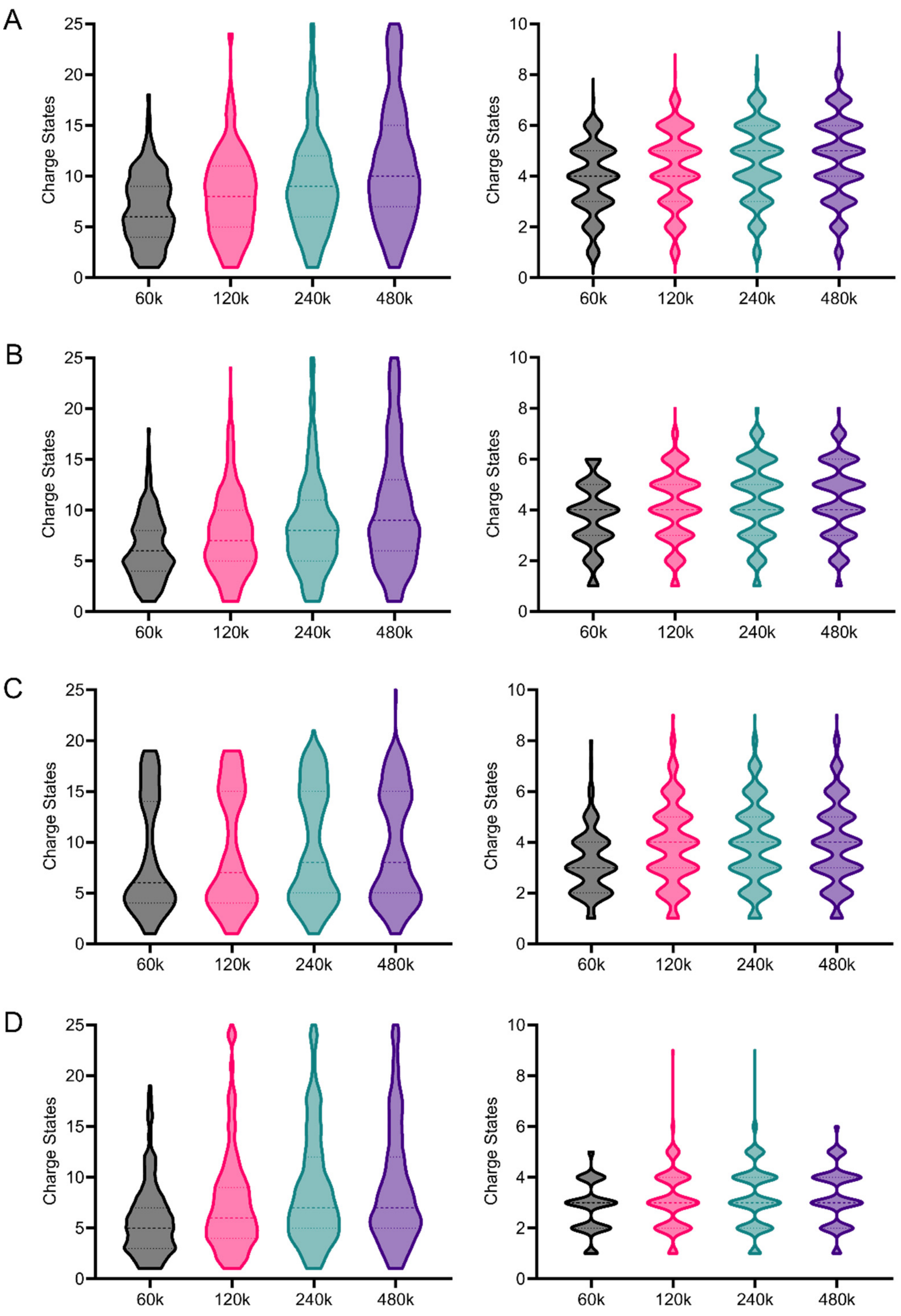

Figure S6. Charge state distribution violin plots of Carbonic anhydrase for (A) ETD, (B) EThcD, (C) HCD, and (D) UVPD fragmentation techniques. Each panel compares MS^2 (left) and PTCR MS^3 (right) results. Experiments performed at higher resolving powers (240,000 in green and 480,000 in purple) exhibit a greater number of high charge state identifications.

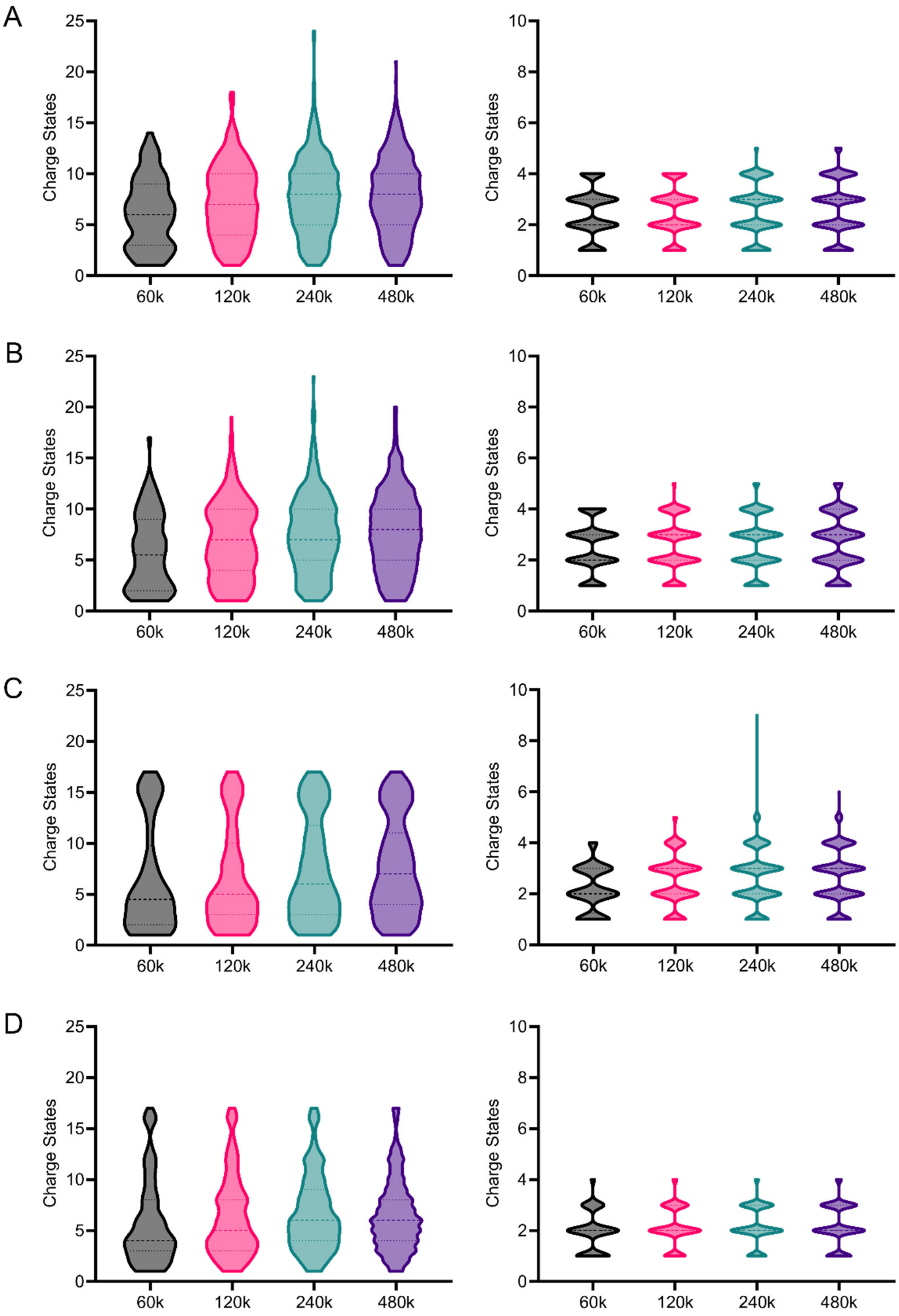

Figure S7. Charge state distribution violin plots of Myoglobin for (A) ETD, (B) EThcD, (C) HCD, and (D) UVPD fragmentation techniques. Each panel compares MS^2 (left) and PTCR MS^3 (right) results. Experiments performed at higher resolving powers (240,000 in green and 480,000 in purple) exhibit a greater number of high charge state identifications.

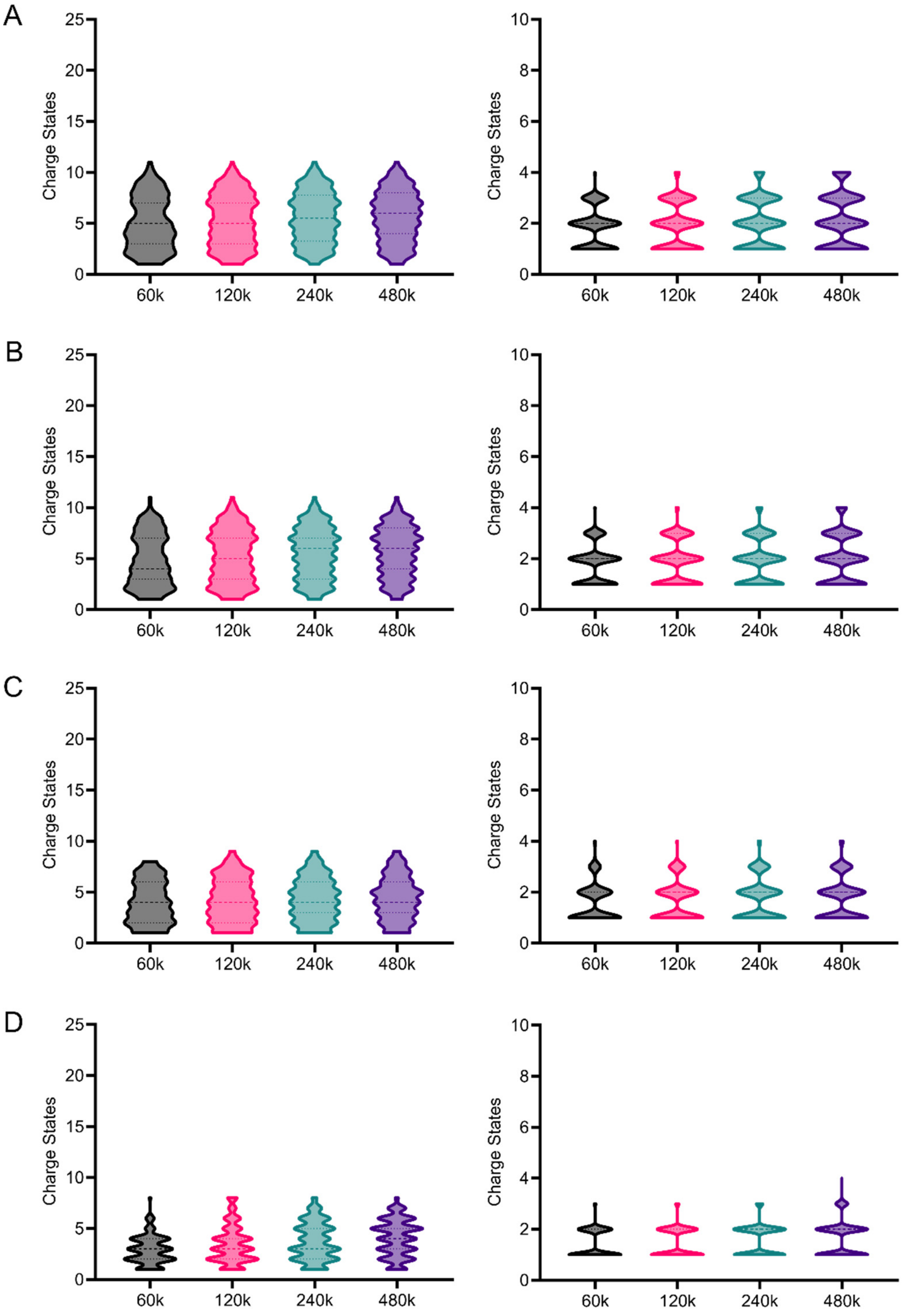

Figure S8. Charge state distribution violin plots of Ubiquitin for (A) ETD, (B) EThcD, (C) HCD, and (D) UVPD fragmentation techniques. Each panel compares MS^2 (left) and PTCR MS^3 (right) results.

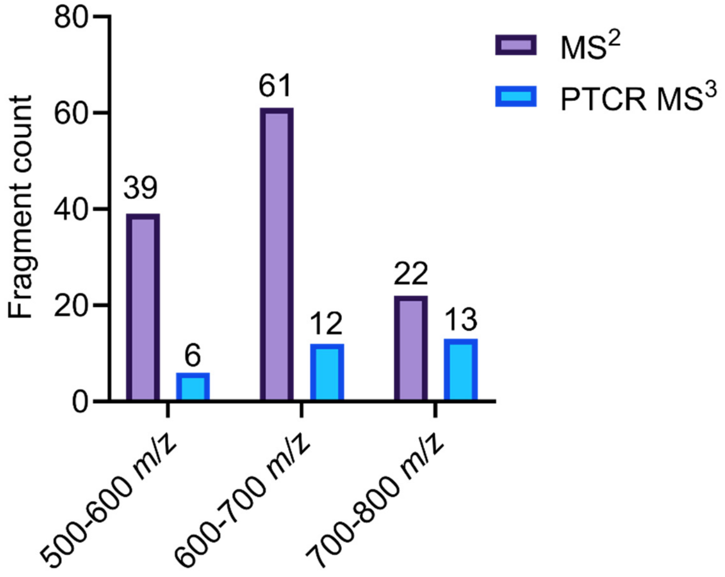

Figure S9. Comparison of fragment ion density between MS^2 and PTCR MS^3 experiments using manually validated carbonic anhydrase ETD data. Fragment counts were evaluated within 100 *m/z* windows across the spectra.

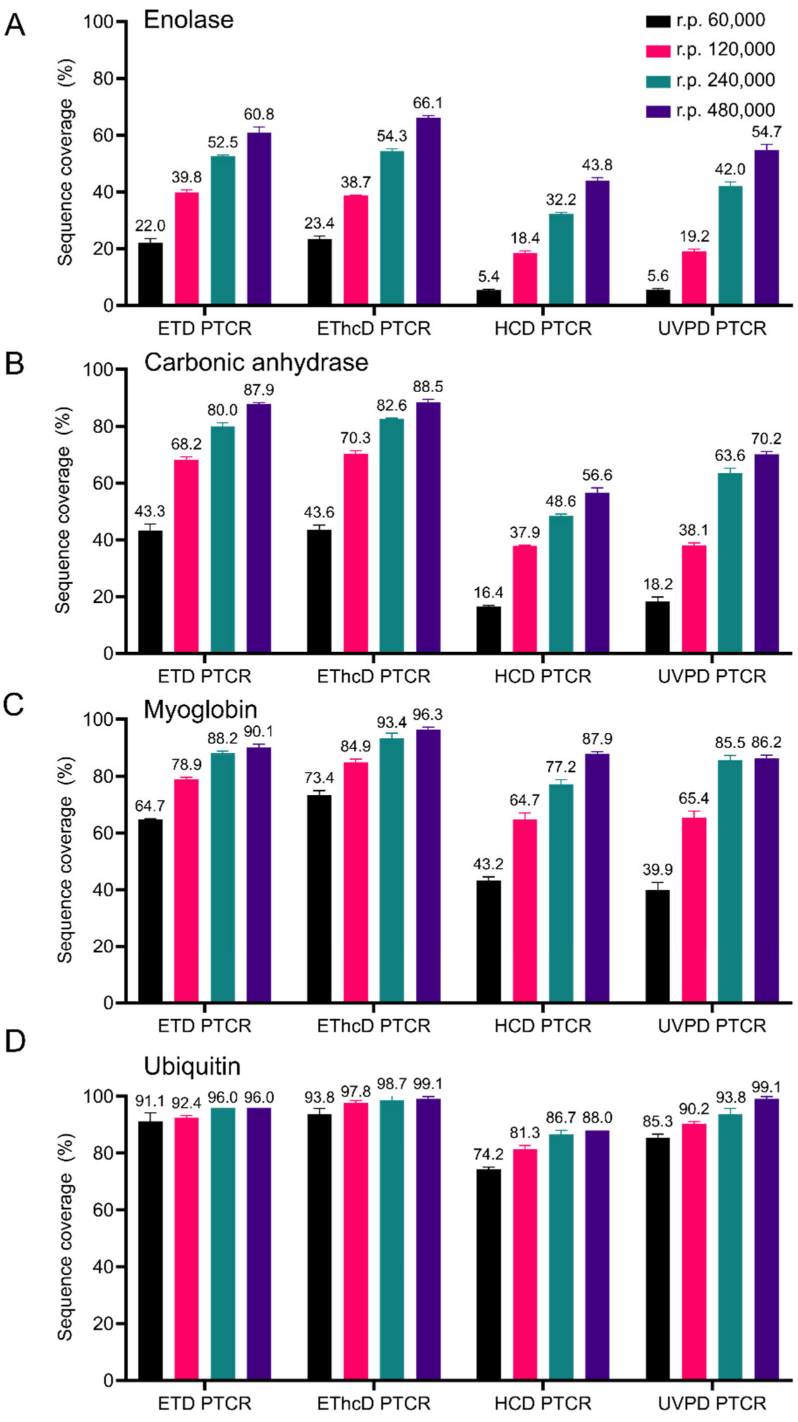

Figure S10. Sequence coverage of (A) Enolase, (B) Carbonic anhydrase, (C) Myoglobin, and (D) Ubiquitin PTCR MS^3 experiments at 60,000, 120,000, 240,000, and 480,000 resolving power in the colors black, pink, green, and purple, respectively. Proteins are arranged in order of molecular weight, with the largest on top (Enolase, 46.6 kDa) and the smallest on the bottom (Ubiquitin, 8.6 kDa).

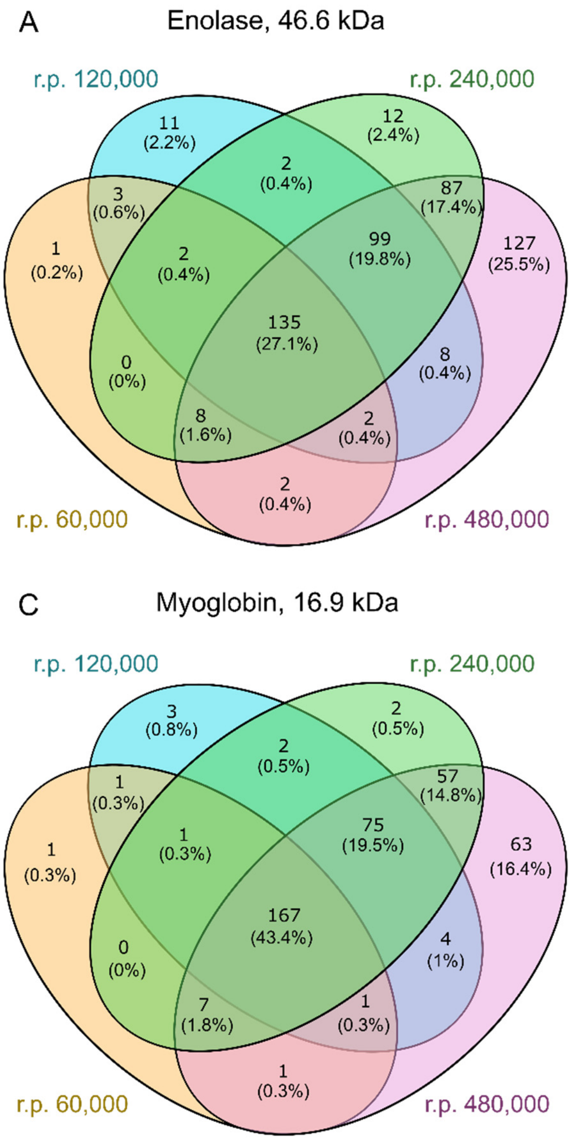

Figure S11. 4-way Venn diagram of unique fragments found in (A) enolase, (B) carbonic anhydrase, (C) myoglobin, and (D) ubiquitin using EThcD MS[2]-PTCR MS[3] at the four resolving powers. The percentage of shared fragments among all resolving powers is shown to increase as protein size decreases.

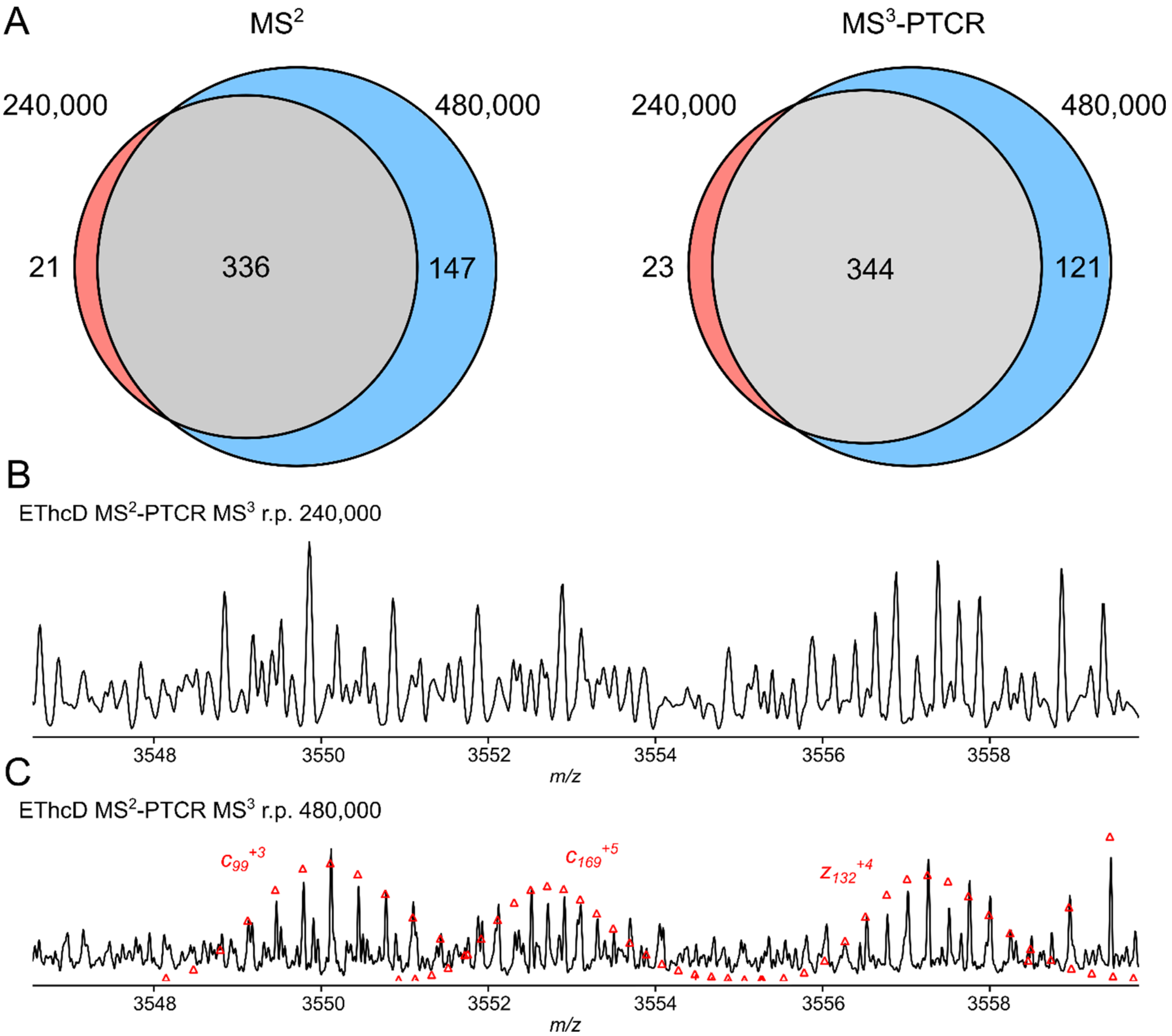

Figure S12. Comparison of unique EThcD fragments identified at different resolving powers in Enolase protein. (A) Venn diagrams of uniquely identified peptides at 240,000 and 480,000 resolving power with (right) and without (left) PTCR MS^3. (B) EThcD MS^2-PTCR MS^3 collect at r.p 240,000 and (C) r.p. 480,000. Three unique fragment ions, c_{99}^{+3}, c_{169}^{+5}, and z_{132}^{+4}, only identified at the r.p. 480,000.

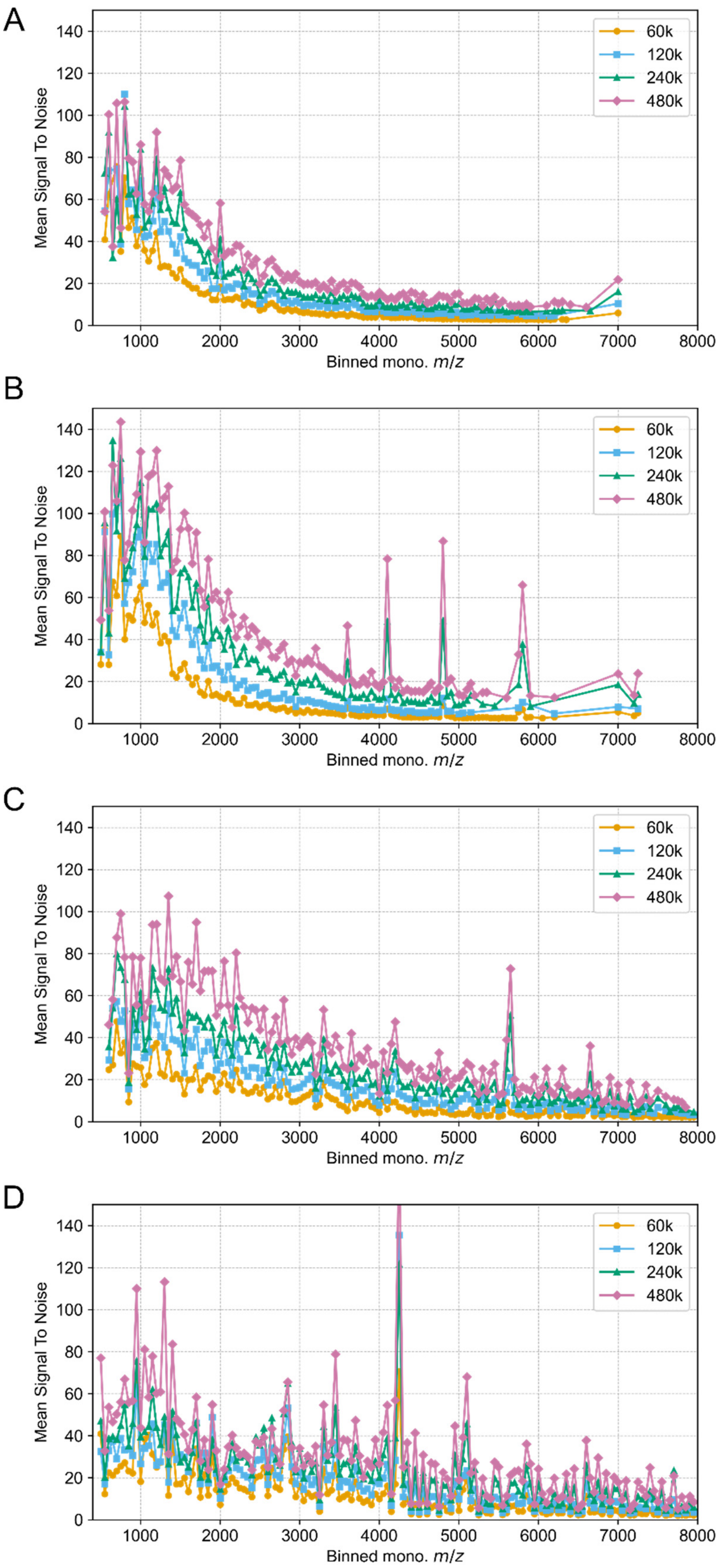

Figure S13. Average signal-to-noise ratio (S/N) distribution across the *m/z* space for (A) enolase, (B) carbonic anhydrase, (C) myoglobin, and (D) ubiquitin in ETD MS^2 – PTCR MS^3 experiments. S/N was directly extracted from RAW files and averaged into 50 *m/z* wide bins. Only the top 10,000 signals were included.

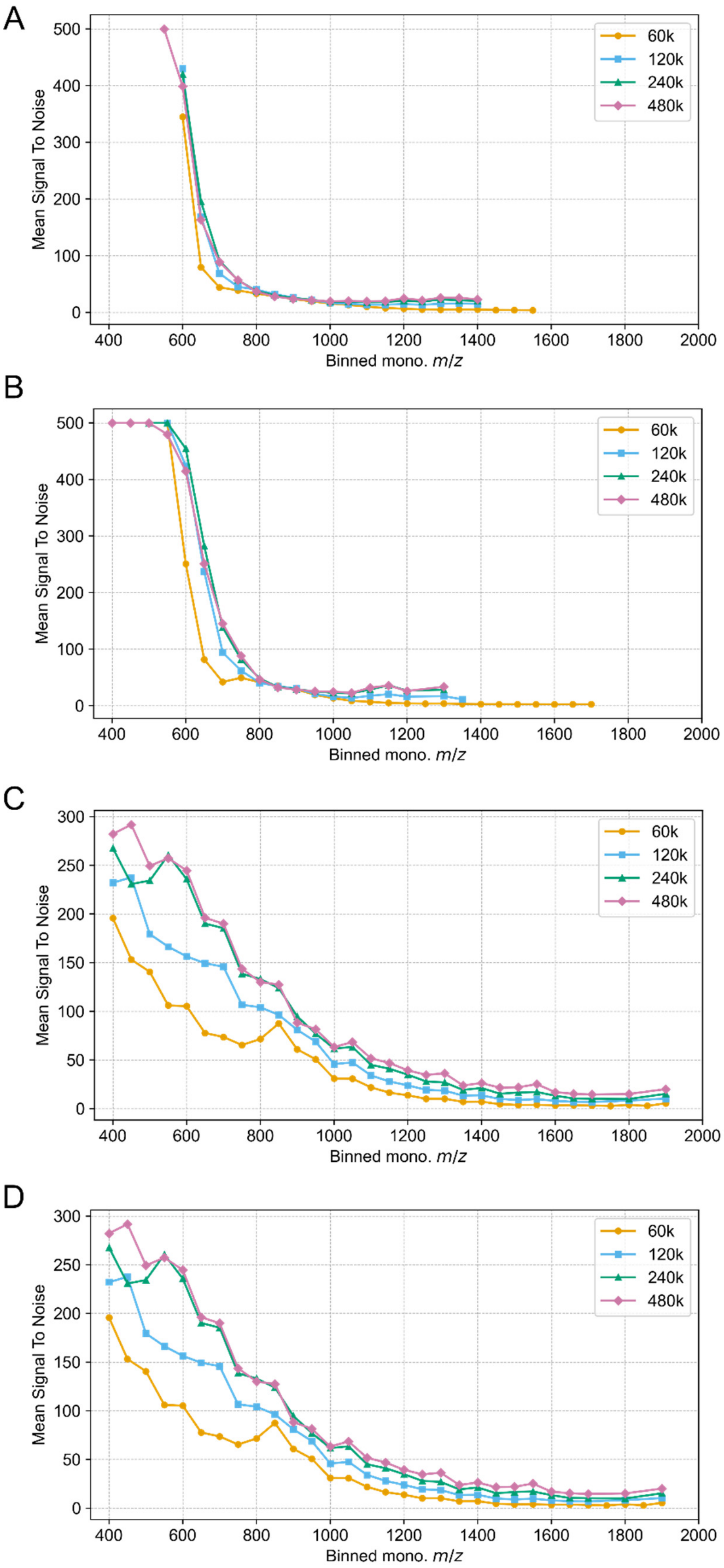

Figure S14. Average signal-to-noise ratio (S/N) distribution across the m/z space for (A) enolase, (B) carbonic anhydrase, (C) myoglobin, and (D) ubiquitin in ETD MS^2 experiments. S/N was directly extracted from RAW files and averaged into 50 m/z wide bins. Only the top 10,000 signals were included. S/N values were capped at 500, and all negative values were removed for purposes of visualization.

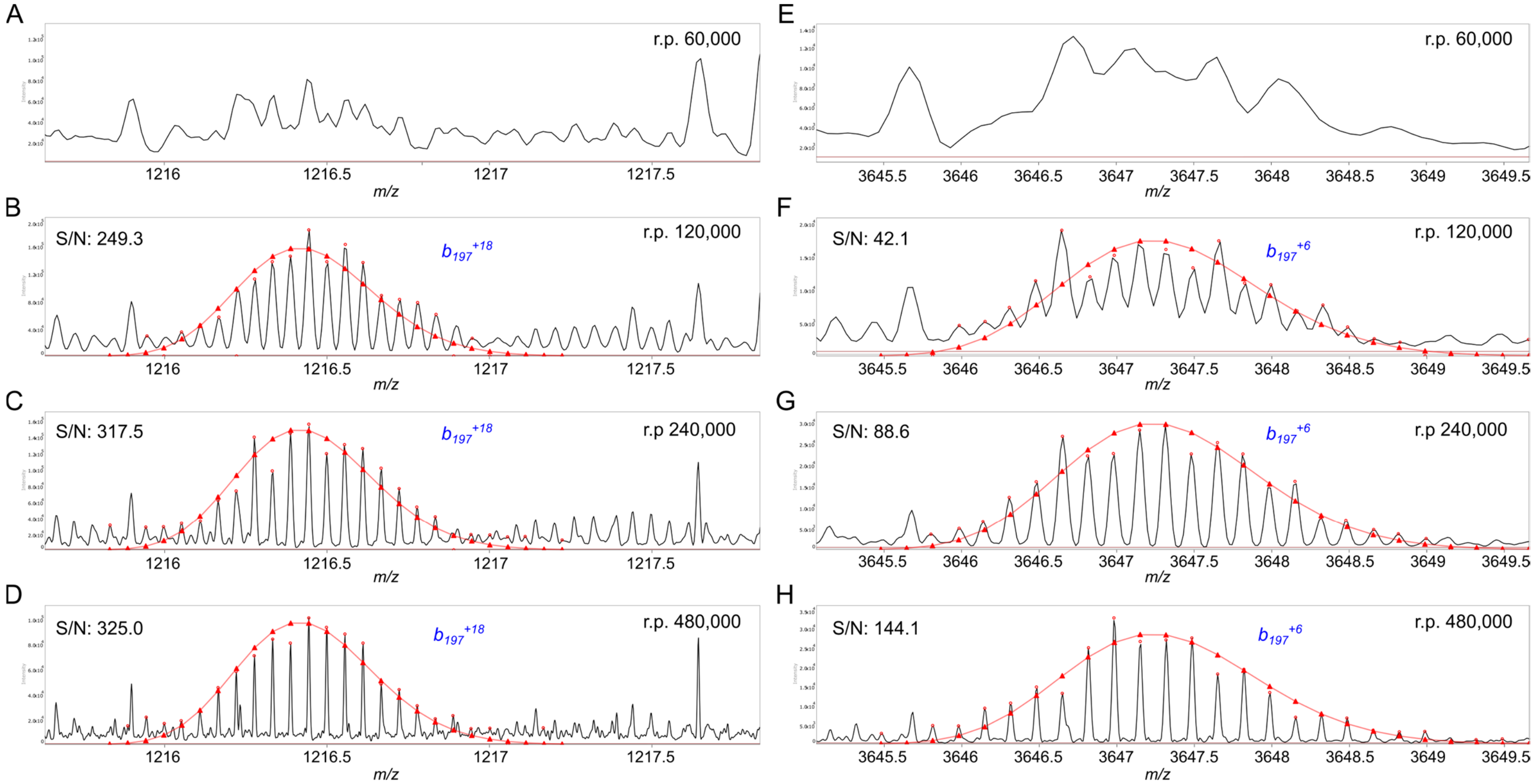

Figure S15. Isotopic distribution and S/N of b_{197} fragment from carbonic anhydrase at HCD MS² (A-D) and PTCR MS³ (E-H) experiments at various resolving powers.

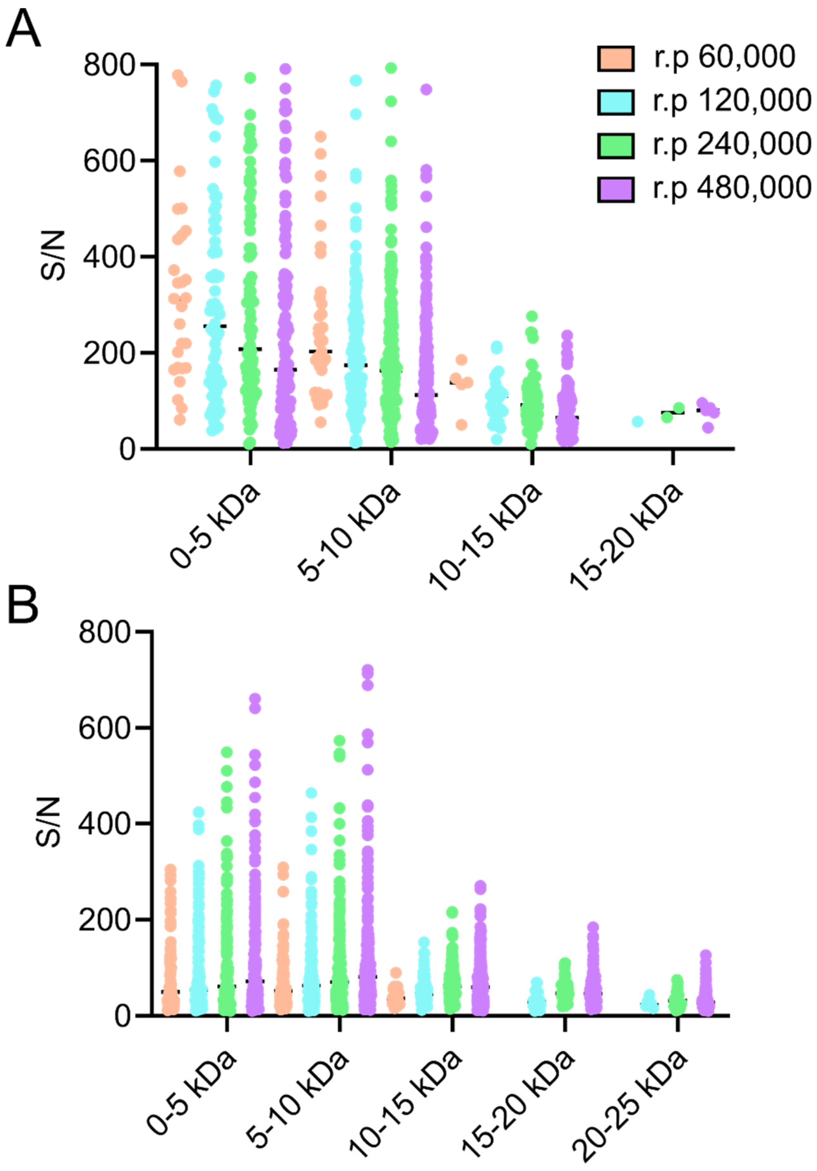

Figure S16. Scatterplot of product ion S/N values grouped into 5 kDa-wide mass bins for (A) MS^2 and (B) PTCR MS^3 results from ETD fragmentation of carbonic anhydrase.

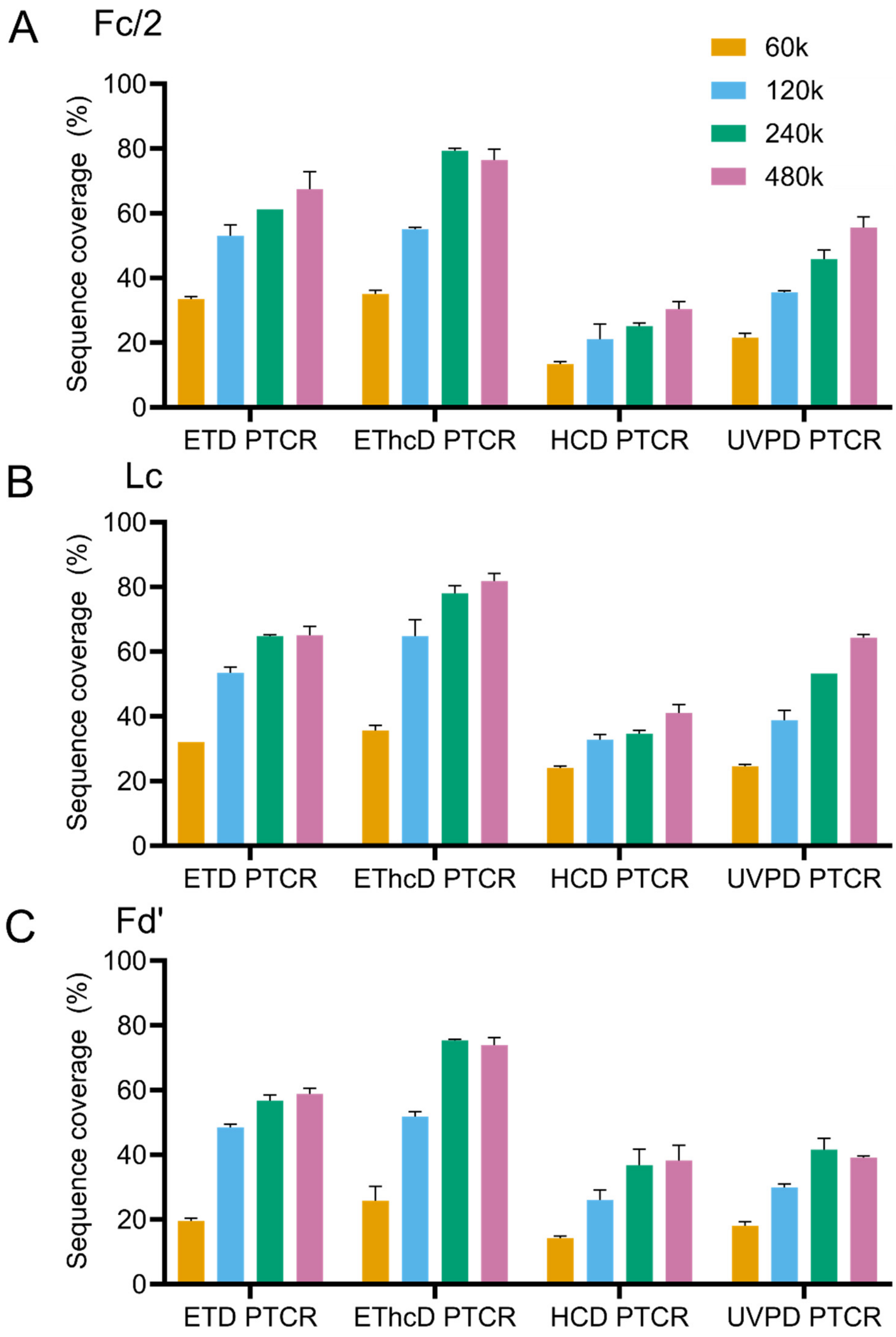

Figure S17. Sequence coverage of NIST mAb subunits (A) Fc/2, (B) Lc, and (C) Fd’ PTCR MS^3 experiments at 60,000, 120,000, 240,000, and 480,000 resolving powers in the colors orange, blue, green, and pink, respectively.

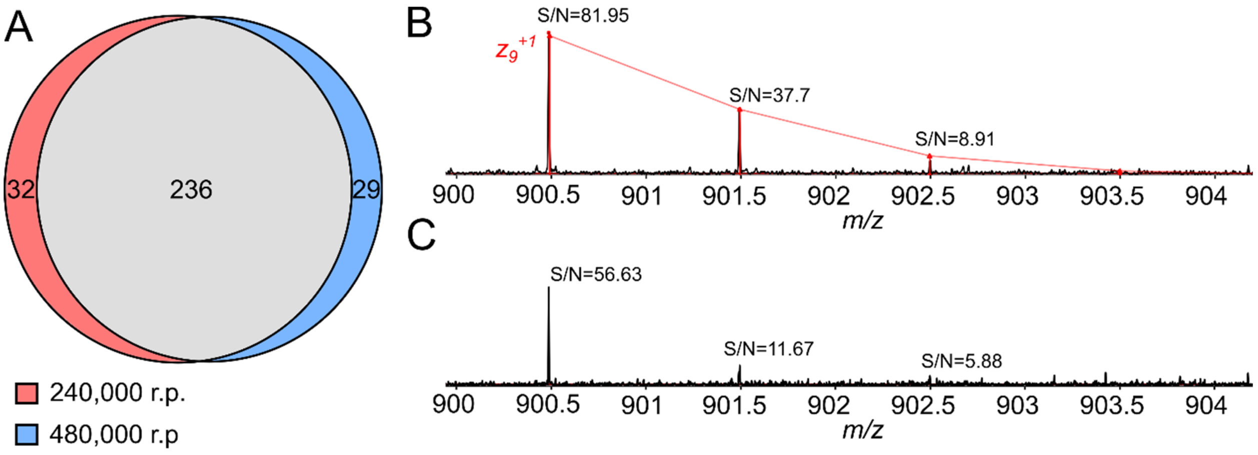

Figure S18. Comparison of unique EThcD fragments identified at different resolving powers for NIST Fc/2 subunit. (A) Venn diagrams of uniquely identified fragments at 240k and 480k resolution with PTCR MS^3. (B) Automatically matched z_9^{+1} fragment ion at 240,000 r.p., (C) not matched at 480,000 r.p. S/N values for isotope peaks were extracted from FreeStyle.
